# Single-molecule imaging of UvrA and UvrB recruitment to DNA lesions in living *Escherichia coli*

**DOI:** 10.1038/ncomms12568

**Published:** 2016-08-26

**Authors:** Mathew Stracy, Marcin Jaciuk, Stephan Uphoff, Achillefs N. Kapanidis, Marcin Nowotny, David J. Sherratt, Pawel Zawadzki

**Affiliations:** 1 Department of Biochemistry, University of Oxford, South Parks Road, Oxford OX1 3QU, UK; 2 Biological Physics Research Group, Clarendon Laboratory, Department of Physics, University of Oxford, Parks Road, Oxford OX1 3PU, UK; 3 Laboratory of Protein Structure, International Institute of Molecular and Cell Biology, 4 Ksiecia Trojdena Street, 02-109 Warsaw, Poland

## Abstract

Nucleotide excision repair (NER) removes chemically diverse DNA lesions in all domains of life. In *Escherichia coli*, UvrA and UvrB initiate NER, although the mechanistic details of how this occurs *in vivo* remain to be established. Here, we use single-molecule fluorescence imaging to provide a comprehensive characterization of the lesion search, recognition and verification process in living cells. We show that NER initiation involves a two-step mechanism in which UvrA scans the genome and locates DNA damage independently of UvrB. Then UvrA recruits UvrB from solution to the lesion. These steps are coordinated by ATP binding and hydrolysis in the ‘proximal' and ‘distal' UvrA ATP-binding sites. We show that initial UvrB-independent damage recognition by UvrA requires ATPase activity in the distal site only. Subsequent UvrB recruitment requires ATP hydrolysis in the proximal site. Finally, UvrA dissociates from the lesion complex, allowing UvrB to orchestrate the downstream NER reactions.

## Abstract (web summary)

Nucleotide excision repair is able to identify and remove a wide range of DNA helix distorting lesions from the genome. Here the authors use single molecule imaging of UvrA and UvrB molecules and suggest a two-step ‘scan and recruit' model for UvrA function.

## Graphical abstract

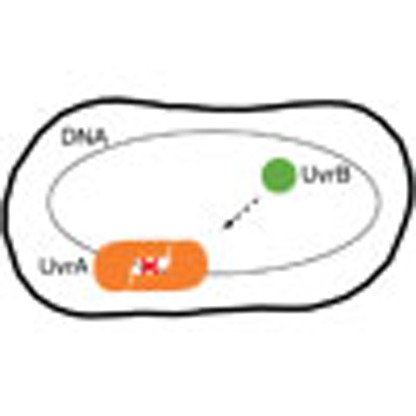

Nucleotide excision repair (NER), safeguards genomes against toxic and mutagenic DNA damage caused by various sources, ranging from ultraviolet light to chemical mutagens^1^,^2^,^3^,^4^. NER is unique among DNA repair mechanisms in its ability to remove a broad spectrum of structurally unrelated DNA lesions^5^. In bacteria, the multistep NER pathway begins with lesion recognition by the UvrA and UvrB proteins, followed by incision at either side of the damage site by UvrC, and removal of the damaged DNA strand by UvrD^3^,^4^,^5^. The resulting gap is then filled in by DNA polymerase 1 and sealed by DNA ligase^3^,^4^,^5^.

*In vitro* studies using purified proteins, including crystallographic studies of NER intermediates^6^,^7^,^8^, have provided a mechanistic framework of the initial steps in the NER pathway^3^,^4^,^5^,^6^,^7^,^8^,^9^,^10^. According to currently accepted models, dimeric UvrA forms a stable complex with either one or two UvrB molecules^3^,^4^,^5^,^10^,^11^. This UvrA–UvrB damage sensor scans the genome to locate DNA lesions. After a lesion is encountered, UvrA dissociates from the complex and leaves UvrB stably bound at the damage site. The UvrA dimer belongs to the ATP-binding cassette family of ATPases^12^, but unlike the majority of the family members, UvrA has two ATP-binding sites per monomer, designated ‘proximal' and ‘distal'^6^,^7^,^8^. The activity of both sites is required for NER, and it was demonstrated that both sites work co-operatively^13^. Furthermore, ATP binding was shown to increase UvrA dimer stability^14^,^15^,^16^ and ATP hydrolysis was implied to be required for UvrA dissociation from DNA^17^. Nevertheless, the precise role of each site, and how these sites co-operate to orchestrate NER remains unclear.

Here, we have used photoactivated localization microscopy (PALM)^18^ alongside single-particle tracking^19^, to analyse the behaviour of the proteins initiating NER in live *Escherichia coli*. By studying the intracellular mobility and positioning of UvrA and UvrB proteins, complemented with *in vitro* assays, we show that UvrA scans the genome, performing two distinct types of DNA interactions, and locates DNA damage independently of UvrB. UvrA recruits UvrB to a lesion only after initial damage detection and verification. Using catalytic mutants of UvrA, we demonstrate a role for ATP binding and hydrolysis in the individual ATP-binding sites. After damage recognition, which requires ATP binding to the distal site, ATP hydrolysis in the proximal site is necessary for UvrB recruitment to DNA. Subsequently, ATP hydrolysis in the distal site is needed for progression of NER. We propose that ATP hydrolysis in the distal site facilitates release of UvrA from the repair complex, allowing UvrB to further verify the presence of damage and coordinate the downstream steps of NER.

## Results

### *In vivo* characterization of UvrA

**Figure 1:**
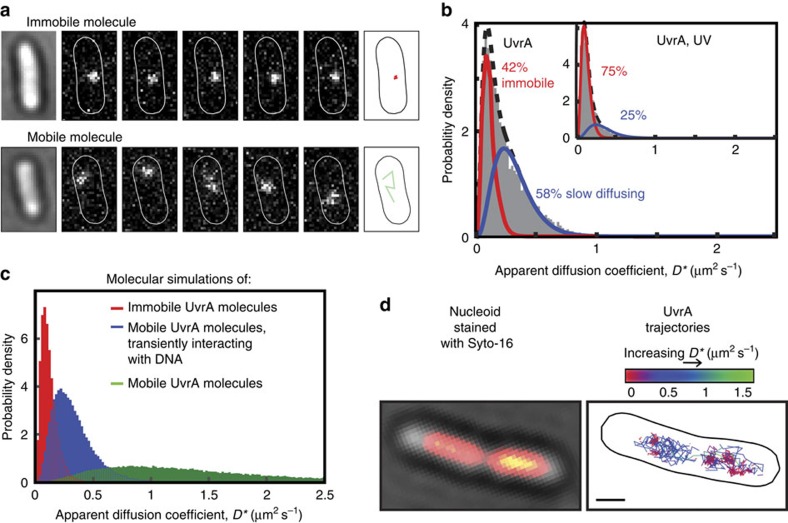
*In vivo* characterization of UvrA. (**a**) Example image of a single immobile UvrA-PAmCherry molecule imaged, localized and tracked at 15 ms exposures over five consecutive frames (top). Example consecutive images showing fast diffusing UvrB-PAmCherry molecule (bottom). (**b**) Distribution of apparent diffusion coefficients (D*) of 8,720 tracked UvrA molecules, fitted with a two species model; an immobile, DNA-bound population (42%±2%, error indicates s.e.m. of three experimental repeats; constrained at *D*_imm_=0.11 μm^2^ s^−1^) and a mobile population of slowly moving molecules (58%±2%; unconstrained fit converged to *D*_slow_=0.31±0.01 μm^2^ s^−1^, *D* value range represents 95% confidence intervals). (Inset) Distribution of D* values of 6,941 tracked UvrA molecules after exposure to 50 J m^−2^ ultraviolet light (UV), fitted with a two species model. (**c**) Distribution D* values of trajectories generated from Monte Carlo diffusion simulations within a typical *E. coli* cell volume. Each simulated trajectory is averaged over 15 ms frame times and 40 nm localization uncertainty added, and the resulting localizations analysed as for experimental data. The D* distribution for simulated immobile molecules is shown in red, and the distribution of D* values expected for freely diffusing UvrA dimers is shown in green. The D* distribution generated from simulations of molecules rapidly interconverting between free diffusion and transient DNA binding is shown in blue (see Supplementary Methods for details). (**d**) Imaging of UvrA molecules and Syto-16-stained DNA in the same cells. To increase nucleoid-free regions, cells were treated with nucleoid-compacting antibiotic chloramphenicol. For analysis of >100 unperturbed cells, and cells after UV exposure, see Supplementary Fig. 1e. Scale bar, 1 μm.

To understand the behaviour of individual UvrA molecules in living cells, we replaced the endogenous *uvrA* gene with a fully functional C-terminal fusion to the photoactivable fluorescent protein, PAmCherry (Supplementary Fig. 1a). Sparse photoactivation of PAmCherry allowed imaging and tracking individual dimeric UvrA molecules with 15 ms exposures, one molecule per cell at a time^19^,^20^. The mobility of each tracked molecule provides a measure of its activity. DNA-bound molecules appear immobile, while diffusing molecules appear mobile and show clear displacements between successive frames^20^,^21^,^22^ (Fig. 1a). These differences in mobility can be quantified by calculating an apparent diffusion coefficient (D*) for each molecule^20^,^22^. The distribution of D* values was poorly described by fitting with the analytical expression for a single diffusing species^21^,^22^ (Supplementary Fig. 1d). Instead, the distribution agreed well with a model for two distinct molecular species^21^,^22^, involving an immobile, DNA-bound population (42% at 0.11 μm^2^ s^−1^; note that the small apparent mobility is due to the localization uncertainty, see Methods) and a mobile population of slowly diffusing molecules (58% at 0.31 μm^2^ s^−1^; Fig. 1b). The slowly diffusing molecules had a lower mobility than expected for freely diffusing UvrA dimers, based on their size (Fig. 1c and Supplementary Methods). This is likely a result of a combination of three-dimensional diffusion and repeated transient interactions with DNA (shorter than the exposure time of 15 ms) during the lesion search process, a conclusion that was supported by Monte Carlo diffusion simulations (Fig. 1c) (ref. 22). To test if slowly diffusing molecules interact with DNA, we tracked UvrA molecules in cells in which DNA was stained with Syto-16, and observed that tracks of slowly diffusing UvrA were nearly always located within the nucleoid region (Fig. 1d, see Supplementary Fig. 1e for analysis >100 cells), in agreement with previous conventional fluorescence microscopy of UvrA^23^. Therefore, we propose that UvrA undergoes multiple transient interactions with DNA during single exposures (15 ms) and that these interactions represent the initial search process.

**Figure 2:**
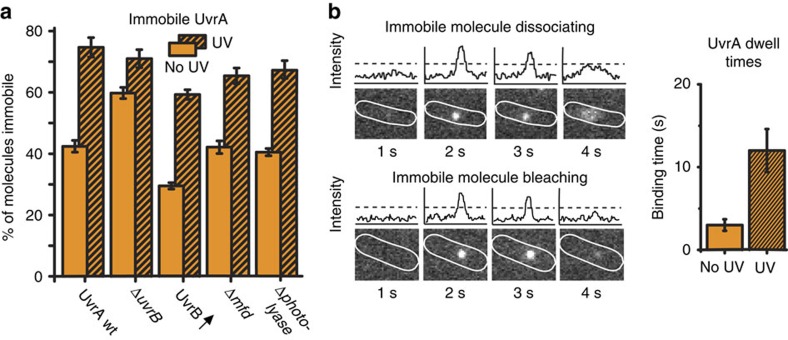
Immobile UvrA molecules. (**a**) Fractions of immobile UvrA molecules extracted by fitting two species model to the UvrA D* distribution before and after exposure to ultraviolet light (UV) in WT cells, Δ*uvrB* cells, cells overexpressing unlabelled UvrB from a plasmid, Δ*mfd* and Δ*photolyase* cells. Errors represent s.e.m. of three experimental repeats. See Supplementary Fig. 2 for fitted D* distributions. (**b**) Dwell time assay. Example cells imaged with 1 s exposures, showing an immobile, DNA-bound UvrA molecule and the corresponding intensity. The UvrA molecule dissociates from DNA (left, top), or is photobleached before dissociation (left, bottom). Photobleaching lifetimes were measured independently in fixed cells, and used to correct for the effects of truncation by photobleaching; see Methods. Binding time distributions for immobile UvrA±UV exposure after correction for photobleaching in WT cells (right). UvrA-binding times were measured with 1 s exposures. See Supplementary Fig. 3 for fitted distributions.

In the absence of exogenous DNA damage, ∼40% of UvrA molecules remained immobile during the entire trajectory. This indicates another step in the lesion search process, involving longer-lasting interactions with non-damaged DNA, distinct from the transient interactions described above. We investigated if these immobile UvrA molecules depend on UvrB. In fact, deletion of *uvrB* resulted in a larger proportion of immobile UvrA molecules than in wild-type (WT) cells, demonstrating that UvrA can bind DNA without UvrB (Fig. 2a and Supplementary Fig. 2). Since Δ*uvrB* cells are non-functional in NER, the increase in immobile UvrA molecules may represent an accumulation on unrepairable spontaneous damage sites. Alternatively, UvrB may facilitate the dissociation of UvrA from non-damaged DNA. This latter conclusion is supported by work of others showing that UvrB decreases the nonspecific DNA-binding activity of UvrA^24^. In agreement with this, when we ectopically overexpressed UvrB, the proportion of immobile UvrA molecules was lower than in cells with normal UvrB expression (Fig. 2a).

UvrA can directly bind DNA, or be recruited to DNA via the transcription-coupling factor Mfd, which binds to stalled RNA polymerase^25^. An interaction has also been implied between UvrA and the DNA repair enzyme, photolyase^26^. We asked whether a proportion of the immobile UvrA molecules in undamaged cells were a consequence of either Mfd or photolyase association. Deletion of *mfd* or *phr* (encoding photolyase) caused essentially no change in the abundance of immobile molecules (Fig. 2a). Since deletion of these known binding partners had no effect on the ∼40% of UvrA molecules that are immobile in undamaged cells, we conclude that the majority of these molecules are directly associated with DNA.

To further understand this immobile population, we measured the time constant of UvrA dissociation from DNA (hereafter referred to as dwell time) of UvrA on DNA in cells. We used low excitation intensities and long exposure times (1 s), such that the fluorescence from mobile molecules was motion-blurred, whereas immobile, DNA-bound, molecules appeared as diffraction-limited spots^13^,^20^,^27^ (Fig. 2b). In the absence of damage, the dwell time of immobile UvrA-PAmCherry, after correction for photobleaching^20^, was 3±0.7 s (Fig. 2b and Supplementary Fig. 3). We interpret this dwell as the time required for the damage verification process when UvrA binds to undamaged DNA (see Discussion).

Inducing DNA damage by exposing cells to ultraviolet light (50 J m^−2^) before PALM imaging caused an increase in the fraction of immobile UvrA molecules (75%, Fig. 1b; inset). After exposure, UvrA still showed strong co-localization with DNA, and we found no evidence of recruitment to the inner cell membrane, as reported previously^28^ (Supplementary Fig. 1e). We asked whether UvrA association with DNA lesions depends on UvrB. In Δ*uvrB* cells, exposure to ultraviolet light still resulted in a significant (*P*=0.03) increase in the fraction of immobile UvrA molecules, to a similar level as seen in WT cells (72%, Fig. 2a). Therefore, UvrA can still detect and bind damaged DNA even in the absence of UvrB. This result agrees with previous *in vitro* observations that UvrA is able to discriminate damaged and undamaged DNA independently of UvrB^6^,^8^,^17^,^29^.

Exposing Δ*mfd* or Δ*phr* cells to ultraviolet light still caused immobilization of UvrA molecules, albeit to slightly lower levels compared with WT cells (Fig. 2a). Both Mfd and photolyase thus show a modest decrease in damage recognition efficiency, consistent with the contribution of these factors to NER initiation^25^,^26^. The dwell time of immobile UvrA was increased to ∼12 s after exposure to ultraviolet light (Fig. 2b), indicating that the coordination of subsequent NER steps takes a longer time than the initial binding events observed for UvrA in the absence of ultraviolet light induced damage. To test if UvrA-binding times are dictated by the availability of UvrB molecules, we overexpressed UvrB and observed that binding times, both in the presence and absence of damage, were mildly reduced (Supplementary Fig. 3b).

### UvrB is rarely complexed with UvrA in solution

**Figure 3:**
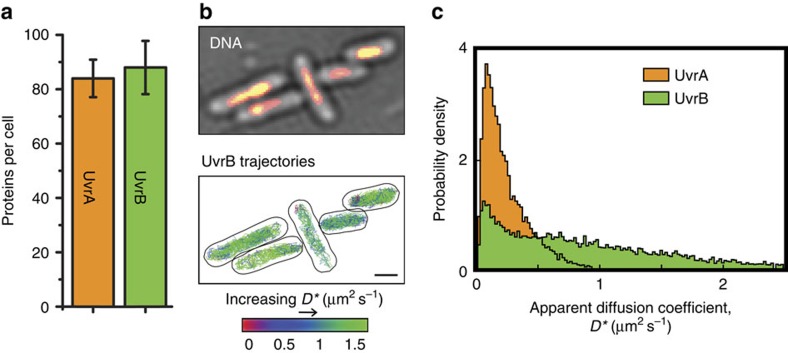
UvrA and UvrB have a different mobility and distribution in cells. (**a**) Mean copy numbers for UvrA and UvrB monomers from >500 cells. No damage was introduced. Errors indicate s.e.m. of three experimental repeats. (**b**) Imaging of UvrB molecules and Syto-16-stained DNA in the same cells. Image represents conditions without introduction of damage. To increase nucleoid-free regions, cells were treated with nucleoid-compacting antibiotic chloramphenicol. Scale bar, 1 μm. For analysis of >100 cells without chloramphenicol treatment, both before and after exposure to ultraviolet light, see Supplementary Fig. 1e. (**c**) Overlay of UvrA and UvrB D* distributions.

**Figure 4:**
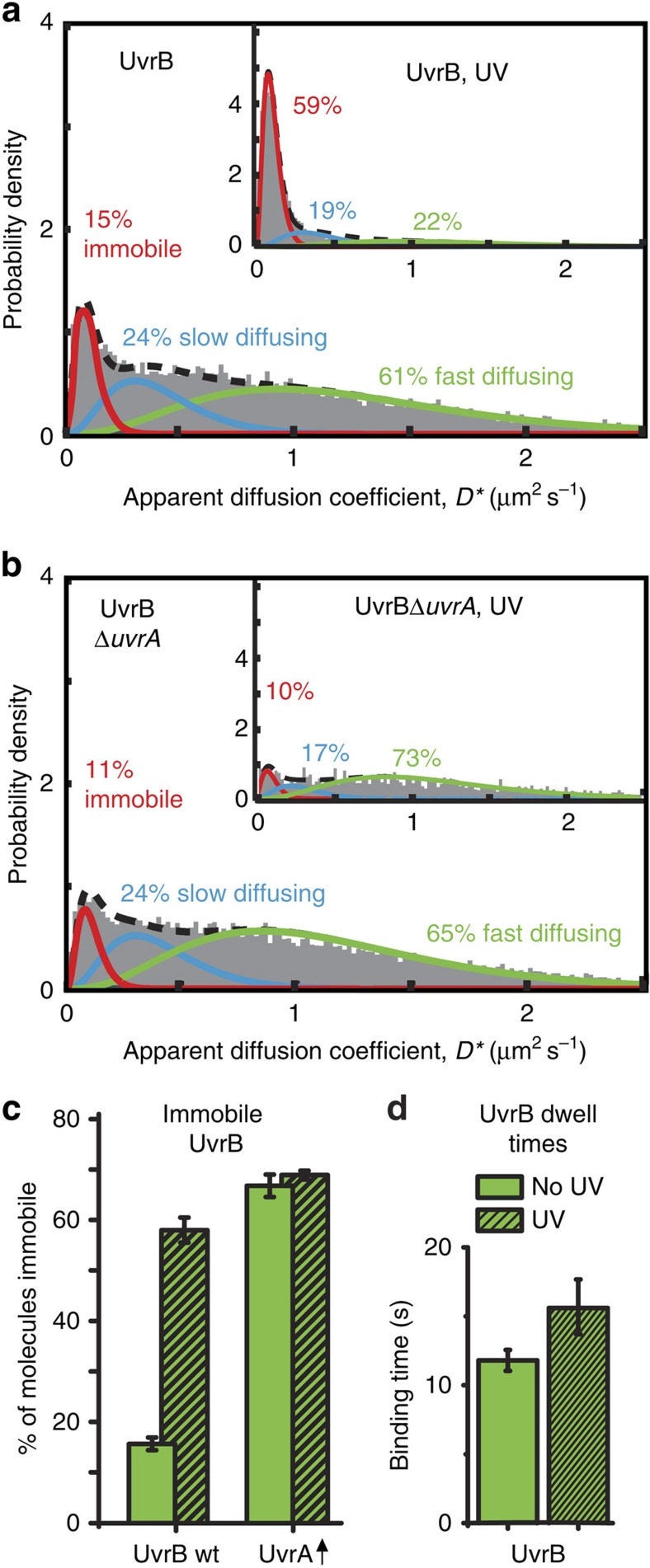
*In vivo* characterization of UvrB. (**a**) Distribution of D* values of 9,958 tracked UvrB molecules, fitted with a three species model (constrained *D*_imm_=0.11 μm^2^ s^−1^) established that 15%±2% of UvrB molecules were immobile, 24%±3%, diffusing slowly (*D*_slow_=0.41±0.02 μm^2^ s^−1^) and 61%±5% fast diffusing (*D*_fast_=1.24±0.02 μm^2^ s^−1^). (Inset) The distribution of D* values of 7,472 tracked UvrB molecules after exposure to ultraviolet light (UV). (**b**) Distribution of D* values of 9,456 tracked UvrB molecules in Δ*uvrA* cells, fitted with a three species model (constrained at D* values obtained for UvrB in WT cells; 0.11, 0.41 and 1.24 μm^2^ s^−1^) established that 11%±3% of UvrB molecules were immobile, 24%±4% diffusing slowly and 65%±7% fast diffusing. (Inset) The distribution of D* values of 1,606 tracked UvrB molecules after exposure to UV. (**c**) Fraction of immobile UvrB molecules extracted by fitting a three species model to the distributions of D* values for WT cells and cells overexpressing unlabelled UvrA. Errors represent s.e.m. from fits to three experimental repeats. (**d**) Dwell time distributions for immobile UvrB±UV after correction for photobleaching. To measure the UvrB binding in the absence of DNA damage we used a strain overexpressing unlabelled UvrA, resulting in recruitment of UvrB to non-damaged DNA. Dwell times were measured with 1 s exposures followed by 4 s delay. Errors represent s.e.m. of three experimental repeats.

To compare the behaviour of UvrA with its partner, UvrB, we replaced the endogenous *uvrB* gene with a fully functional C-terminal PAmCherry fusion (Supplementary Fig. 1a), and imaged using the same protocol as for UvrA. Because current models propose that NER is initiated by a stable complex of UvrA and UvrB^3^,^4^,^5^,^8^,^9^,^10^, and our data showed that UvrB is present at a similar level to UvrA (both ∼85 copies per cell; Fig. 3a and Supplementary Fig. 1b,c), the models predict similar diffusion profiles and spatial distributions for UvrA and UvrB. This was not the case; in contrast with UvrA, UvrB molecules were located throughout the cell volume, with relatively little bias towards nucleoid regions (Fig. 3b, for analysis of >100 cells see Supplementary Fig. 1e). Moreover, the distribution of apparent diffusion coefficients of UvrB molecules was very different from that of UvrA. The majority of UvrB molecules showed a much higher mobility than any UvrA molecules, and only a small fraction of UvrB molecules were immobile or slow moving (Fig. 3c). Consequently, a model for two molecular species described the D* distribution of UvrB poorly (Supplementary Fig. 1f). On the other hand, fitting an analytical model with three molecular species matched the data well and established that 15% of UvrB molecules were immobile (0.11 μm^2^ s^−1^), 24% were diffusing slowly (0.41 μm^2^ s^−1^) and 61% were fast diffusing (1.24 μm^2^ s^−1^; Fig. 4a). Since UvrA did not have a fast moving population, the fast diffusing UvrB molecules (accounting for ∼60% of the total) cannot be complexed with UvrA, as demonstrated in the non-overlapping regions of the two distributions in Fig. 3c. Although a quarter of UvrB molecules were slowly diffusing, their mobility did not match with that of the mobile UvrA molecules (D*=0.41±0.01 and 0.31±0.02 μm^2^ s^−1^, respectively; *P*<0.01 *t*-test; see Methods). This indicates that these slow-moving populations may not represent UvrA–UvrB complexes. To further test this, we imaged UvrB-PAmCherry in Δ*uvrA* cells, and detected no significant change to the abundance (*P*=0.96 *t*-test) or mobility (*P*=0.75 *t*-test) of slowly diffusing UvrB molecules (compare Fig. 4b with Fig. 4a). Therefore, we conclude that *in vivo*, UvrA and UvrB rarely form a complex in solution.

In WT cells, exposure to ultraviolet light caused relatively little change to the abundance of slowly diffusing UvrB, however, it caused a large increase in the abundance of immobile molecules (59%, Fig. 4a, inset), together with a concomitant decrease in the population of fast diffusing molecules. This is consistent with recruitment of UvrB directly from solution to damage sites. In contrast, in Δ*uvrA* cells no such UvrB recruitment was observed after ultraviolet light exposure (Fig. 4b). In undamaged Δ*uvrA* cells, we observed only a small reduction (from 15 to 11%) in the abundance of immobile UvrB molecules compared with undamaged WT cells (Fig. 4a). These 4% of UvrA-dependent immobile UvrB molecules in WT cells may represent a low level of basal DNA repair, or occasional recruitment of UvrB as a result of ‘false-positive' damage recognition by UvrA. Indeed, imaging UvrB-PAmCherry in undamaged cells ectopically overexpressing unlabelled UvrA caused a large increase in the immobile UvrB population, showing that UvrA can recruit UvrB to non-damaged DNA (Fig. 4c and Supplementary Fig. 2e). The level of this ‘false-positive' or spontaneous UvrB recruitment in WT cells remains to be established.

To probe the duration of the damage verification process, without the subsequent repair steps, we exploited the ability to recruit UvrB to undamaged DNA by overexpressing UvrA. We found that the dwell time of immobile UvrB in undamaged cells overexpressing UvrA was 11.8±0.8 s (Fig. 4d and Supplementary Fig. 3c). In WT cells, after exposure to ultraviolet light, the dwell time was 15.6±1.9 s. This is longer than the UvrA-binding time after exposure to ultraviolet light (12±2.6 s; Fig. 2d), consistent with models in which UvrA dissociation precedes recruitment of UvrC by UvrB^3^,^5^,^10^. When compared with the dwell times of proteins involved in the final stages of DNA repair (DNA polymerase 1, 2.1 s; ligase, 2.5 s)^20^ the initial steps of NER were slow.

### UvrA efficiently recruits UvrB from solution *in vitro*

The observations that UvrA and UvrB are rarely complexed in solution *in vivo*, and that UvrA dimers can locate damage sites independently of UvrB, suggest that the current models, in which a stable UvrA–UvrB complex scans the genome to initiate NER^3^,^4^,^5^,^7^,^8^,^9^,^10^, need revision. We propose that NER initiation is a two-step process, where UvrA performs initial damage recognition and verification, and recruits UvrB from solution to perform further damage verification.

**Figure 5:**
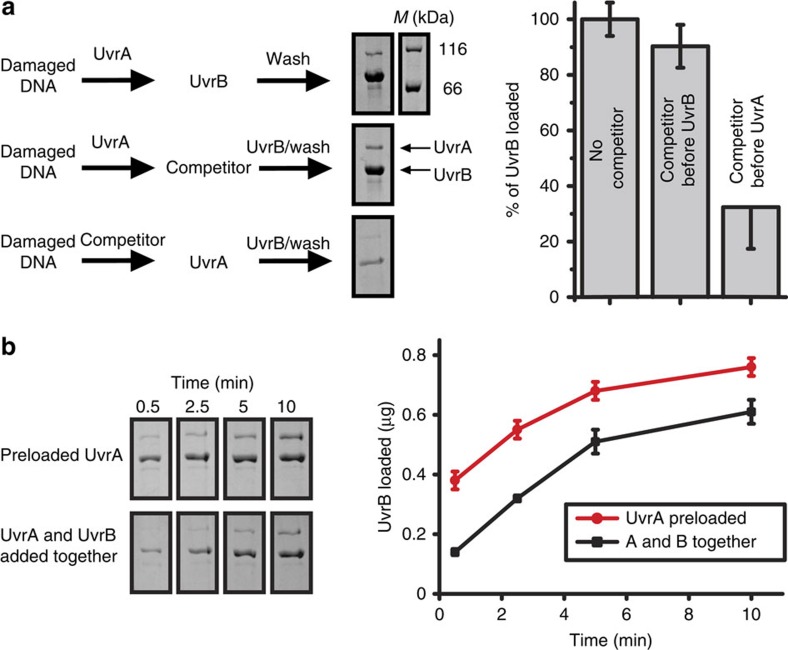
*In vitro* UvrB-loading assays. (**a**) Competition assay. UvrA was incubated with biotinylated DNA, containing a site-specific fluorescein lesion, attached to magnetic beads. Subsequently, UvrB was added and the amount of UvrB recruited to damaged DNA beads was quantified. As a competitor, a 10-fold excess of damaged DNA containing the same site-specific lesion was added to the reaction, either before addition of UvrA or before addition of UvrB. Images of representative gels are presented and mean of three independent experiments is shown on the right. The total amount of UvrB protein loaded onto the beads was quantified and compared between the three conditions used. The most efficient loading reaction was set as 100%. Errors represent s.d. of three experimental repeats. For full gel images see Supplementary Fig. 4. (**b**) Time course experiment, which monitored the efficiency of UvrB recruitment to damaged DNA over time. Two reactions were performed with either UvrA preloaded onto the damaged DNA followed by UvrB at *t*=0, or premixed UvrA and UvrB were added together at *t*=0. Images of representative gels are presented and the mean of three independent experiments is shown on the right. Errors represent s.e.m. of three experimental repeats. For full gel images see Supplementary Fig. 4.

To verify our *in vivo* observations, we reconstituted a UvrB recruitment reaction *in vitro* using pull-down experiments with magnetic beads conjugated to damaged DNA. We preloaded UvrA onto the damaged DNA and asked if UvrB could be recruited by DNA-bound UvrA or if UvrA must first dissociate and form a UvrA–UvrB complex in solution. UvrA rebinding was prevented by introducing a large excess of an unconjugated damaged DNA competitor. Introduction of the competitor before adding UvrA to the damaged DNA resulted in a ∼65% reduction in UvrB loading, compared with a control where no competitor was used (Fig. 5a). On the other hand, introduction of the competitor after addition of UvrA, but before addition of UvrB, resulted in comparable recruitment of UvrB compared with the no-competitor control (Fig. 5a and Supplementary Fig. 4a). This demonstrates that UvrA does not need to dissociate from the damage site and that it can load UvrB onto the lesion directly from solution.

To compare the efficiency of UvrB recruitment by preloaded UvrA versus loading via UvrA–UvrB complexes preformed in solution, we performed a time-course experiment under two conditions and quantified the amount of UvrB recruited to damaged DNA. At time point zero, premixed UvrA and UvrB were added to the damaged DNA, and in a parallel experiment, UvrB alone was added to the damaged DNA, which had been preloaded with UvrA. We found that addition of UvrB to preloaded UvrA increased the efficiency of UvrB loading (Fig. 5b and Supplementary Fig. 4b). No UvrB recruitment was observed when UvrA was omitted from the reaction (Supplementary Fig. 4c). This result supports our *in vivo* observations, and further verifies our proposed two-step model, in which UvrA is able to locate and verify DNA damage independently of UvrB, and subsequently recruits UvrB to damage sites directly from solution.

### UvrA ATPase function in damage recognition

**Figure 6:**
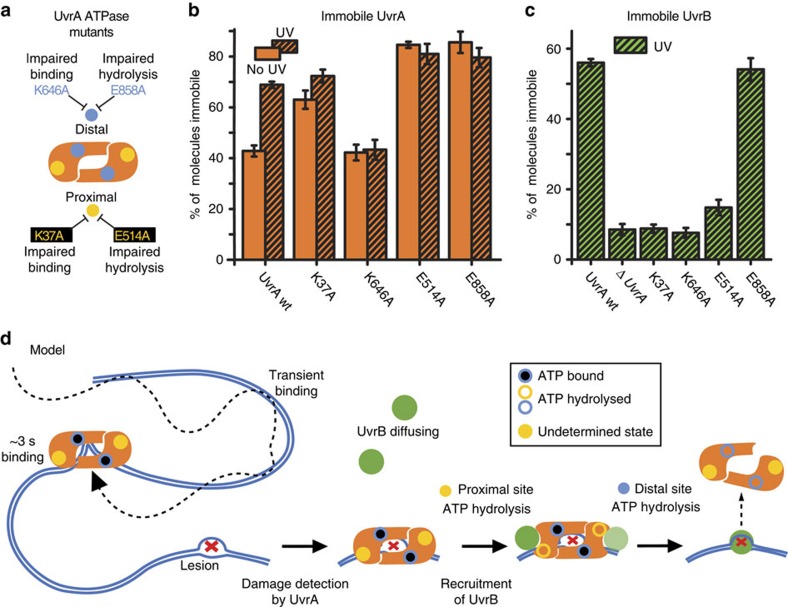
UvrA ATPase is required for recruitment of UvrB. (**a**) Cartoon of the UvrA dimer showing the ATPase mutations used. (**b**) Fraction of immobile UvrA-PAmCherry mutants before and after exposure to ultraviolet light (UV). See Supplementary Fig. 6 for fitted D* distributions. UvrA mutants were expressed from a plasmid in Δ*uvrA*, Δ*mfd* cells. As a control, WT UvrA-PAmCherry expressed from a plasmid was used. Errors represent s.e.m. of three experimental repeats. (**c**) Fraction of immobile UvrB-PAmCherry molecules recruited to DNA after exposure to UV light. Unlabelled UvrA or UvrA mutants were expressed from a plasmid in Δ*uvrA*. See Supplementary Fig. 7 for fitted D* distributions. Errors represent s.e.m. of three experimental repeats. (**d**) Cartoon showing the proposed model for the initiation of NER. Dimeric UvrA, with ATP bound in the distal site, scans the genome for lesions making transient interactions with DNA. At putative lesions (represented as a kink), UvrA performs a damage verification step lasting ∼3 s. After positive damage identification, UvrA hydrolyses ATP in the proximal site to recruit either one or two UvrB molecules to the lesion. Subsequent hydrolysis of the distal site ATP facilitates the release of UvrA from the pre-incision complex.

UvrA monomers have two ATP-binding sites; proximal and distal^6^,^8^ (Fig. 6a and Supplementary Fig. 5), and catalytic mutants impaired in ATP binding in either site are defective in NER^13^,^17^,^29^ (Supplementary Fig. 6f). The presence of two ATPase sites in one protein suggests that UvrA might play a role in coordinating a cascade of NER steps, however the functions of the individual sites remain to be fully understood^13^,^16^,^17^. Imaging the catalytic mutant, UvrA^K37A^-PAmCherry (proximal site impaired in ATP binding) showed an increase in the abundance of immobile molecules compared with WT UvrA. A similar behaviour was observed for WT UvrA in Δ*uvrB* cells (Fig. 2a), which we interpreted as a result of reduced UvrA displacement from DNA by UvrB. Nevertheless, the fraction of immobile UvrA^K37A^ molecules showed a significant increase after exposure to ultraviolet light (Fig. 6b) demonstrating that ATP binding and hydrolysis in the proximal site are not required for initial damage detection by UvrA.

On the other hand, UvrA^K646A^-PAmCherry (distal site impaired in ATP binding) showed a reduction in the fraction of slowly diffusing molecules, and the appearance of a fast diffusing species, indicative of UvrA molecules with reduced DNA binding, that could arise because of weakened UvrA dimerization (Supplementary Fig. 6d). This interpretation is consistent with *in vitro* observations that ATP is important for UvrA dimer stability^14^,^16^,^29^. Exposure to ultraviolet light did not increase the fraction of immobile UvrA^K646A^ molecules (Fig. 6b), showing that ATP binding to the distal site is required for initial damage recognition, possibly by allowing UvrA to adopt the appropriate dimer conformation.

To further investigate the role of ATP hydrolysis in either site, we introduced mutations that support ATP binding, but are impaired in ATP hydrolysis (Supplementary Methods). A mutant with impaired ATP hydrolysis in the proximal site but with a WT distal site (UvrA^E514A^), as well as a mutant with impaired ATP hydrolysis in the distal site but with a WT proximal site (UvrA^E858A^), showed increased fractions of immobile molecules independently of the presence of damage (Fig. 6b). This agrees with previous *in vitro* work using the poorly hydrolysable ATP analogue, ATPγS, which demonstrated that allowing ATP binding, but impairing hydrolysis, promotes tight nonspecific DNA binding of UvrA^17^.

### UvrA ATPase function is required for recruitment of UvrB

Since UvrA can detect damage sites independently of UvrB *in vivo* (Fig. 2a), we addressed whether recruitment of UvrB is dependent on ATP binding or hydrolysis by UvrA. We imaged UvrB-PAmCherry in Δ*uvrA* cells in which UvrA mutant proteins were ectopically expressed. Exposure to ultraviolet light did not cause immobilization of UvrB molecules for either UvrA mutants defective in ATP binding, in contrast to WT UvrA (Fig. 6c and Supplementary Fig. 7). Therefore, impairment of ATP binding in either site of UvrA prevents UvrB recruitment to DNA, in agreement with *in vitro* studies using the same mutations, which found that coordinated action of both UvrA ATPase sites was needed for the delivery of UvrB to damaged DNA^13^,^29^.

To further understand this coordinated behaviour, and to unravel the precise contribution of each ATP-binding site, we analysed the ability of the UvrA ATP-hydrolysis mutants (see above) to recruit UvrB to DNA. Despite being mostly immobile (Fig. 6b), UvrA^E514A^ (impaired ATP hydrolysis in the proximal site) supported only a low level of UvrB recruitment that was not stimulated by ultraviolet light exposure, suggesting that ATP hydrolysis in the proximal site is required for efficient UvrB recruitment (Fig. 6c). In contrast, UvrA^E858A^ (impaired hydrolysis in the distal site), showed efficient recruitment of UvrB to DNA, independent of damage induction (Fig. 6c and Supplementary Fig. 7f). Since blocking ATP binding to the distal site (UvrA^K646A^) prevented loading of UvrB (Fig. 6c), this demonstrates that ATP hydrolysis in the proximal site supports UvrB recruitment, only when ATP binding at the distal site is also permitted. Despite being able to recruit UvrB, UvrA^E858A^ is defective in NER (Supplementary Fig. 6f), showing that a step subsequent to UvrB recruitment requires ATP hydrolysis in the distal site (see Discussion).

## Discussion

In this work we have used photoactivated single-molecule tracking in live *E. coli* cells to gain a new perspective on the initial steps in the NER pathway. On the basis of the very different mobility and spatial organization for UvrA and UvrB proteins, together with perturbations using deletions and overexpression of these proteins, and other binding partners, we propose a two-step model in which the lesion search, recognition and verification of damage is performed by UvrA alone. After positive verification of a putative damage site, UvrA recruits UvrB to perform a second damage verification step, followed by coordination of the downstream NER reactions if required. This was confirmed with *in vitro* assays, which showed that UvrB was more efficiently loaded to DNA damage by UvrA preloaded on DNA, rather than when both proteins were pre-incubated in solution (Fig. 5) Consistent with our conclusion, a previous single-molecule *in vitro* study observed that UvrB can load onto UvrA that is already bound to DNA^11^. In addition, cell fractionation experiments revealed that UvrA is present in the DNA-associated fraction, whereas UvrB was found in the cytoplasmic fraction^28^. Furthermore, this mechanism resolves the apparent conundrum of how Mfd can recruit UvrA via the same binding interface occupied by UvrB in the UvrA–UvrB complex^25^.

Our experiments show that UvrA undergoes two different modes of interaction with DNA while searching for lesions, namely a slowly moving state and a more stably bound state. We propose that slowly moving UvrA scans the genome making only very transient interactions with DNA (<<15 ms). It is interesting to note that the NER initiation factor in yeast (Rad4) uses a ‘twist-open' mechanism to scan the genome, which requires transient (100–500 μs) associations with DNA^30^. In addition, we observed a large population of immobile UvrA molecules associated with undamaged DNA for ∼3 s, consistent with the *in vitro* observation that UvrA binds undamaged DNA for 7 s (ref. 11). Since UvrA recognizes a large variety of chemically diverse lesions, we suggest that this long dwell represents a slow damage verification step involving DNA conformation sampling to distinguish NER substrates from other DNA backbone distortions, such as protein-induced DNA kinks and A-rich DNA stretches^4^. Because there are significantly more immobile UvrA than UvrB molecules in undamaged WT cells, we conclude that only a small proportion of these UvrA molecules proceed to recruit UvrB, and we propose that UvrA rejects the majority of sites it interrogates during these ∼3 s dwells. Nevertheless, we observed that some UvrA molecules can recruit UvrB to undamaged DNA, consistent with an earlier report showing that NER occasionally acts on undamaged DNA^31^. The molecular mechanism used by UvrA to scan the genome and distinguish damaged and undamaged DNA remains to be understood.

By using catalytic mutants of UvrA that fail to bind or hydrolyse ATP, we determined the role of each of its two ATP-binding sites. Our results show that ATPase activity in the proximal site does not play a role in initial damage recognition. However, ATP binding to the distal site is important in this process, likely because it is required for UvrA dimer formation. We therefore propose that UvrA dimers scanning the genome for damage essentially always have ATP bound at the distal site. This is consistent with studies demonstrating that UvrA in solution binds ATP^17^. After damage recognition, ATP hydrolysis in the proximal site is necessary for UvrB recruitment to DNA (Fig. 6). Indeed, structural studies predicted a conformational change after ATP binding and/or hydrolysis in the proximal site, which might expose its UvrB-binding interface^6^,^8^.

In order for UvrB to perform damage verification and recruit UvrC, UvrA must dissociate from the pre-incision complex^3^,^4^,^5^. In agreement with this, our dwell time measurements showed that UvrB remains bound to damaged DNA for ∼16 s, compared with ∼12 for UvrA. However, the mechanism by which UvrA is displaced from the pre-incision complex is not clear. Our observation that UvrA^E858A^ (inhibited hydrolysis in the distal site) can recruit UvrB but is defective in NER (Supplementary Fig. 6f) suggests that a downstream step of NER, following the recruitment of UvrB, is compromised. Since we observed that blocking ATP binding in the distal site might cause dimer destabilization (Supplementary Fig. 6d) we propose that release of UvrA from the pre-incision complex, subsequent to UvrB recruitment, is facilitated by hydrolysis of ATP in the distal site. Consistent with this, imaging UvrA^E858A^-PAmCherry showed a large fraction of immobile molecules even without exposure to ultraviolet light (Fig. 6b), most likely because of a failure of UvrA^E858A^ to dissociate from accumulated spontaneous damage, or from undamaged DNA after false-positive damage recognition. Intriguingly, UvrA^E514A^-PAmCherry also shows a large population of immobile molecules even without damage (despite not being able to recruit UvrB), which points to further coordination between the two ATP-binding sites, yet to be unravelled.

In summary, we have revealed that the early stages of NER involve multiple steps that require coordinated and sequential ATP binding and hydrolysis (Fig. 6d). We have shown that UvrA searches for lesions independently of UvrB. We propose that ATP binding in the distal site is essential for UvrA damage detection. After binding to a lesion, UvrA recruits UvrB from solution, in reaction requiring ATP hydrolysis in the proximal site. Subsequently, UvrA release from the pre-incision complex is triggered by ATP hydrolysis in the distal site, allowing UvrB to coordinate downstream steps of NER (Fig. 6d). The released UvrA can then rapidly form functional dimers again after rebinding ATP in the distal site, and continue its search for lesions.

## Methods

### Bacterial strains and growth

Bacterial strains are listed in Supplementary Table 1. Oligonucleotides and plasmids are shown in Supplementary Table 2 and 3, respectively. Strains were streaked onto Luria–Bertani plates with appropriate antibiotics. Single colonies were inoculated into M9 glycerol (0.2%) and grown overnight at 37 °C to A_600_ 0.4–0.6, then diluted into fresh M9 and grown to A_600_ 0.1. Cells were centrifuged and immobilized on agarose pads between two glass coverslips. We prepared 1% agarose pads by mixing low-fluorescence 2% agarose (Bio-Rad) in dH_2_O 1:1 with 2 × growth medium. DNA damage was introduced by exposing cells to the indicated dose of 254 nm ultraviolet light. Cells were imaged between 5 and 15 min after ultraviolet light exposure. See Supplementary Methods.

### PALM imaging and single-particle tracking

Single-molecule-tracking PALM in live cells was performed using a custom-built total internal reflection fluorescence microscope. Photoactivatable mCherry activation used a 405 nm laser, with excitation at 561 nm. Where indicated DNA was stained with Syto-16 and imaged with a 488 nm laser. Brightfield cell images were recorded with an LED source and condenser (ASI Imaging). PALM single-molecule-tracking analysis used custom-written MATLAB software (MathWorks). We distinguished bound and diffusing proteins by calculating an apparent diffusion coefficient D*=MSD/(4 Δ*t*) from the mean-squared displacement (MSD) for each track with 4 steps at Δ*t*=15 ms. Note that D* is an apparent diffusion coefficient because of cell confinement, motion blurring and localization uncertainty^32^. Immobile molecules have a non-zero D* value due to the ∼40 nm localization uncertainty in each measurement, *σ*_loc_, which manifests itself as a positive offset in the D* value of *σ*_loc_^2^/Δ*t*≈0.1 μm^2^ s^−1^. Significance testing was perform using a *t*-test of D* values extracted from fits to five independent experimental repeats (Supplementary Methods).

### Measuring long-lasting binding events

Long-duration binding events were recorded at low continuous 561 nm excitation intensities using 1 s exposure times. At these exposure times mobile UvrA-PAmCherry and UvrB-PAmCherry molecules are motion blurred over a large fraction of the cell, whereas immobile molecules still appear as point sources, producing a diffraction limited spot. The probability of observing a particular on-time is the product of the underlying binding-time probability and the bleaching probability. The bleaching-time distributions were measured independently using UvrA-PAmCherry in cells fixed with paraformaldehyde, with the same acquisition and excitation conditions. On-time and bleaching-time distributions were fitted with single-exponential functions to extract exponential-time constants *t*_on_ and *t*_bleach_, and the binding-time constant was calculated by *t*_bound_=*t*_on_·*t*_bleach_/(*t*_bleach_−*t*_on_).

### *In vitro* UvrB loading assay

The UvrB loading competition assay was performed with final concentrations of 60 nM UvrA (dimer) and 120 nM UvrB. UvrA was added to a buffer containing 1 mM ATP and 60 nM of biotinylated damaged DNA (50 bp with a fluorescein lesion). Subsequently, magnetic beads (2 mg Dynabeads) were added, incubated then washed. UvrB was added and the reaction was incubated for 2 min at 37 °C with mixing. The reaction was stopped by increasing the NaCl concentration to 1 M. Beads were pelleted with a magnet, washed three times, re-suspended and resolved quantitatively on an SDS–PAGE gel. Two additional reactions were performed with 600 nM of damaged competitor DNA (50 bp with fluorescein lesion, not biotinylated), added either before addition of UvrA or before addition of UvrB.

The time-course assay was performed with the same final concentrations of UvrA and UvrB as above. Two reactions were performed in parallel. In reaction A, UvrA was added to a buffer containing 1 mM ATP and 60 nM of biotinylated damaged DNA. Subsequently, magnetic beads and 600 nM of undamaged DNA (50 bp, not biotinylated) were added. At *t*=0, UvrB was added and the reaction incubated at 37 °C with mixing. The 500 μl samples were taken after 30 s, 2.5 min, 5 min and 10 min, and loading reactions were stopped by increasing the NaCl concentration to 1 M. In reaction B, at *t*=0, premixed UvrA and UvrB were added to a buffer containing the biotinylated damaged DNA, the undamaged DNA, ATP and magnetic beads (Supplementary Methods).

### Data availability

The data that support the findings of this study are available from the corresponding author on request.

## Additional information

**How to cite this article**: Stracy, M. *et al*. Single-molecule imaging of UvrA and UvrB recruitment to DNA lesions in living *Escherichia coli*. *Nat. Commun.* 7:12568 doi: 10.1038/ncomms12568 (2016).

## Supplementary Material

Supplementary Information Supplementary Figures 1-7, Supplementary Tables 1-3, Supplementary Methods and Supplementary References

Peer review file
