# Supplementary material for: Single-molecule imaging of UvrA and UvrB recruitment to DNA lesions in living Escherichia coli
Source: Nat Commun. 2016 Aug 26;7:12568. doi: 10.1038/ncomms12568 (PMC5007444; doi:10.1038/ncomms12568)
Supplement: Supplementary Information — Supplementary Figures 1-7, Supplementary Tables 1-3, Supplementary Methods and Supplementary References [file ncomms12568-s1.pdf]

Supplementary Informations

Supplementary Figures

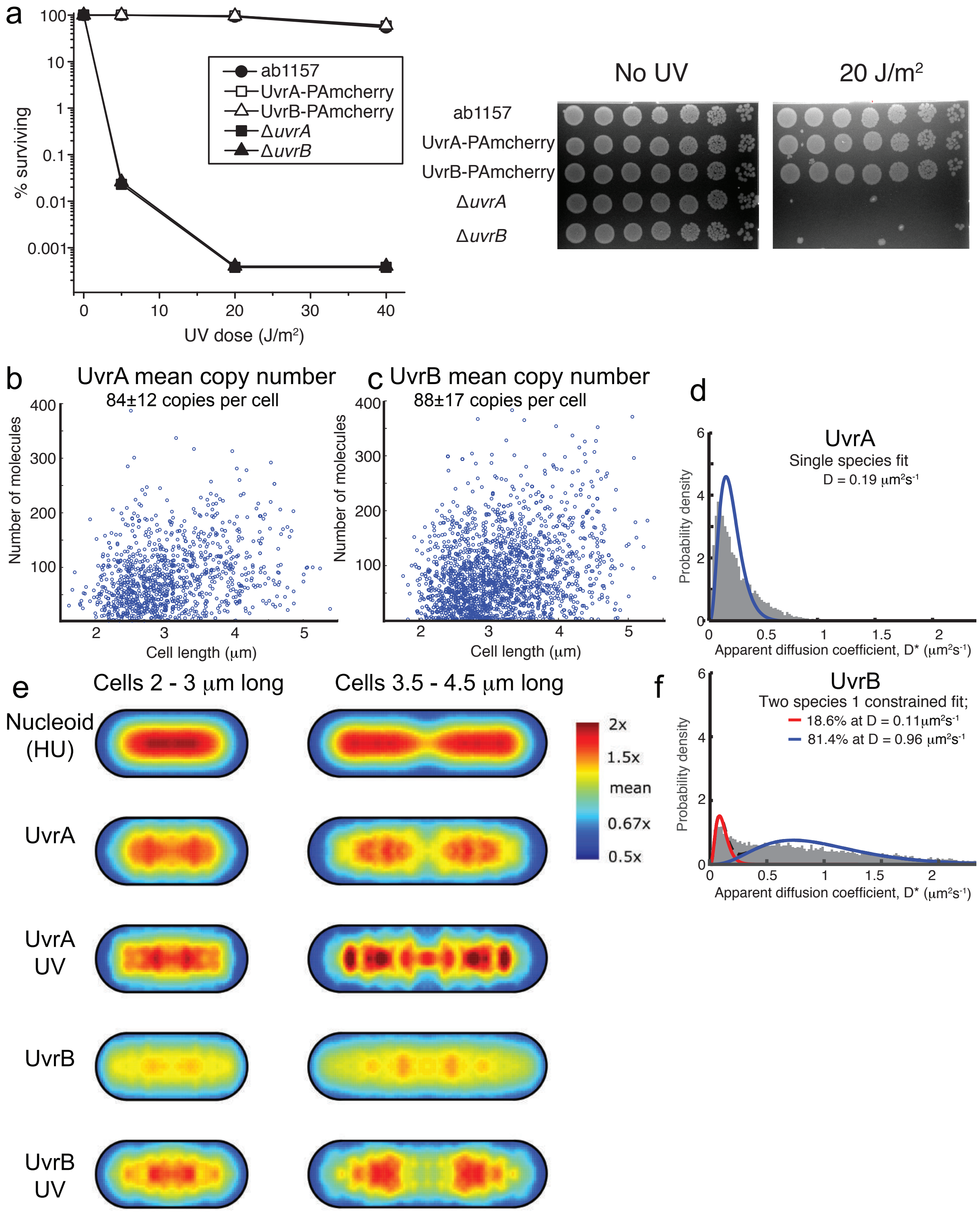

Supplementary Figure 1. UvrA and UvrB fusions to PAmCherry are fully functional. a) UV sensitivity assays. The mean percentage survival of three

experimental repeats as a function of UV dose (left). An example of plates with no UV exposure, and after 20 J/m^2 UV exposure (right). **b**) Copy number distribution for UvrA-PAmCherry molecules against cell length for 1104 cells. No damage was introduced. Our assay counts individual proteins, therefore the copy number relates to copy of UvrA monomers. **c**) Copy number distribution for UvrB-PAmCherry molecules against cell length for 2260 cells. No damage was introduced. **d**) A single species fit to the distribution of UvrA *D** values. The data is poorly described by a single species model (see Fig. 1b for the two species fit). **e**) Normalized 2D histogram plots showing the average spatial distribution of categorized molecules from many cells. Cells in minimal media binned by cell length with short cells (2-3 μm long) having a single centrally located nucleoid, and longer cells (3.5-4.5 μm long) having two clearly separate nucleoids. As a control we used HU-PAmCherry, which is known to associate with nucleoid[1]. UvrA, independently of presence of damage, resulted in similar distribution to observed for HU. This demonstrates that UvrA associates with the nucleoid independently of damage. On the other hand, UvrB showed association with nucleoid only after treatment with UV. UvrA and UvrB have different spatial distribution within living cells supporting our conclusion based on differences in D* distributions that UvrA and UvrB are rarely complexed in solution. Nevertheless, after exposure to UV, UvrB showed much stronger association with the nucleoid demonstrating that these UvrB molecules are performing repair while bound to the nucleoid (see Supplementary Methods for details of 2D normalization). **f**) The distribution of UvrB *D** values is poorly described by a two species model (see Fig. 4a for the three species fit).

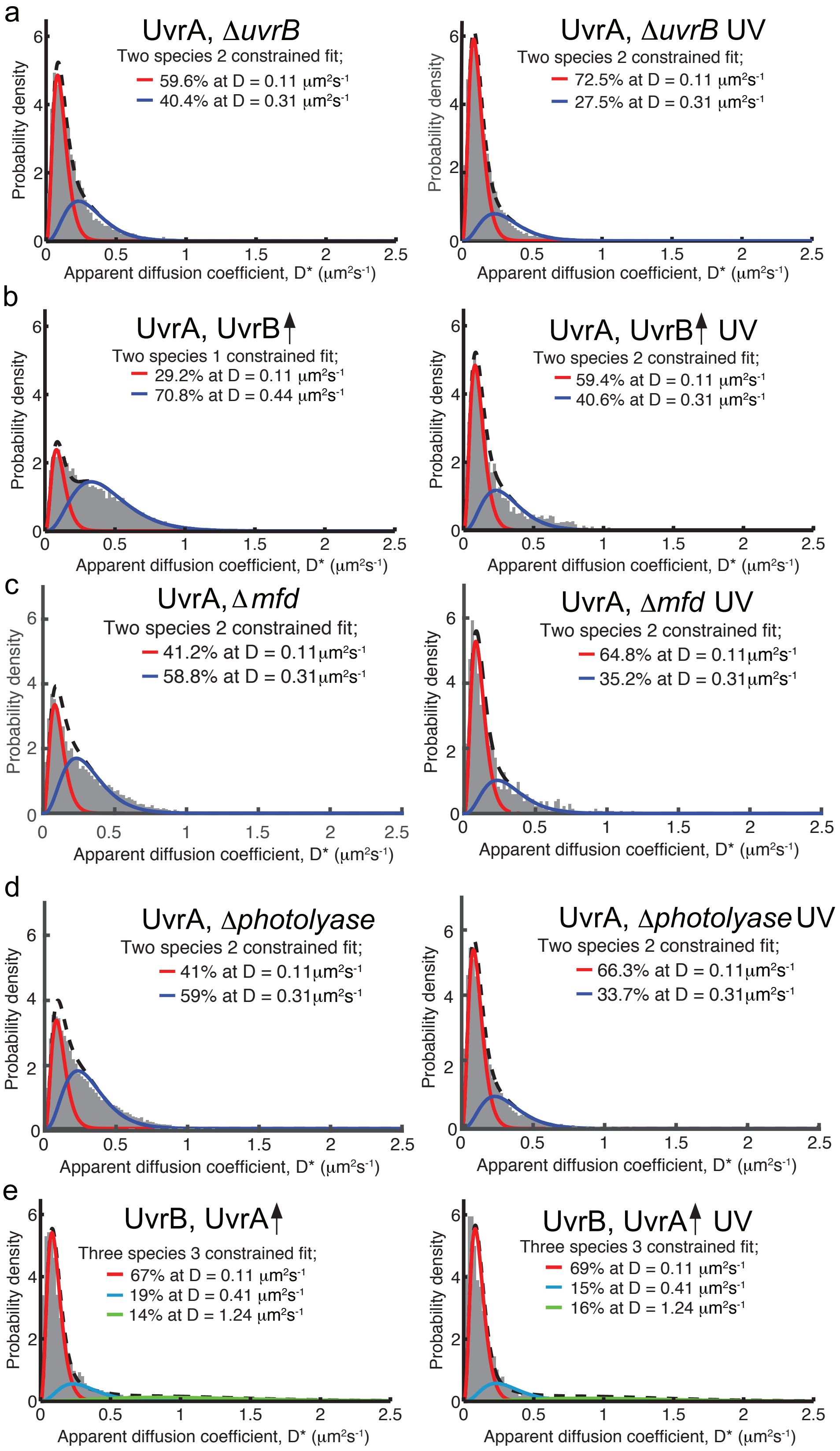

Supplementary Figure 2. Mobility of UvrA and UvrB. a) The distribution of apparent diffusion coefficients (*D*)* for UvrA in a strain with its *uvrB* gene deleted,

without UV exposure (left, 5305 trajectories) and after UV exposure (right, 4371 trajectories). Both D^* values were constrained at values established for wt UvrA (D_{imm} = 0.11 μm^{2}s^{-1}, D_{slow} = 0.31 μm^{2}s^{-1}). **b**) The UvrA D^* distribution in a strain overexpressing unlabelled UvrB (denoted by the arrow), without UV exposure (left, 11278 trajectories) and after UV exposure (right, 1902 trajectories). Fitting the D^* distribution of cells not exposed to UV with a two species model (with D_{imm} = 0.11 μm^{2}s^{-1} constrained, second species unconstrained), showed that the mobile population showed modest increase in mobility compared to in wt cells (D_{slow} = 0.44 μm^{2}s^{-1}). **c**) Distribution of D^* values from 32554 UvrA trajectories in Δ*mfd* cells, fitted with a two species model. Both D^* values were constrained at values established for UvrA in wt cells (D_{imm} = 0.11 μm^{2}s^{-1}, D_{slow} = 0.31 μm^{2}s^{-1}). Right, distribution of D* values from 2027 trajectories in Δ*mfd* cells after exposure to 50 J/m^2 UV, fitted with a two species model. Both D^* values were constrained at values established for UvrA in wt cells (D_{imm} = 0.11 μm^{2}s^{-1}, D_{slow} = 0.31 μm^{2}s^{-1}). **d**) The UvrA D^* distribution in a Δ*photolyase* cells, without UV exposure (left, 12957 trajectories) and after UV exposure (right, 15433 trajectories). The distribution was fitted with a two species model with both values constrained (D_{imm} = 0.11 μm^{2}s^{-1}, D_{slow} = 0.31 μm^{2}s^{-1}). **e**) The UvrB D^* distribution in a strain overexpressing unlabelled UvrA, without UV exposure (left, 5631 trajectories) and after UV exposure (right, 4381 trajectories). The distributions were fitted with a three species model with all three values constrained (D_{imm} = 0.11 μm^{2}s^{-1}, D_{slow} = 0.41 μm^{2}s^{-1}, D_{slow} = 1.24 μm^{2}s^{-1}).

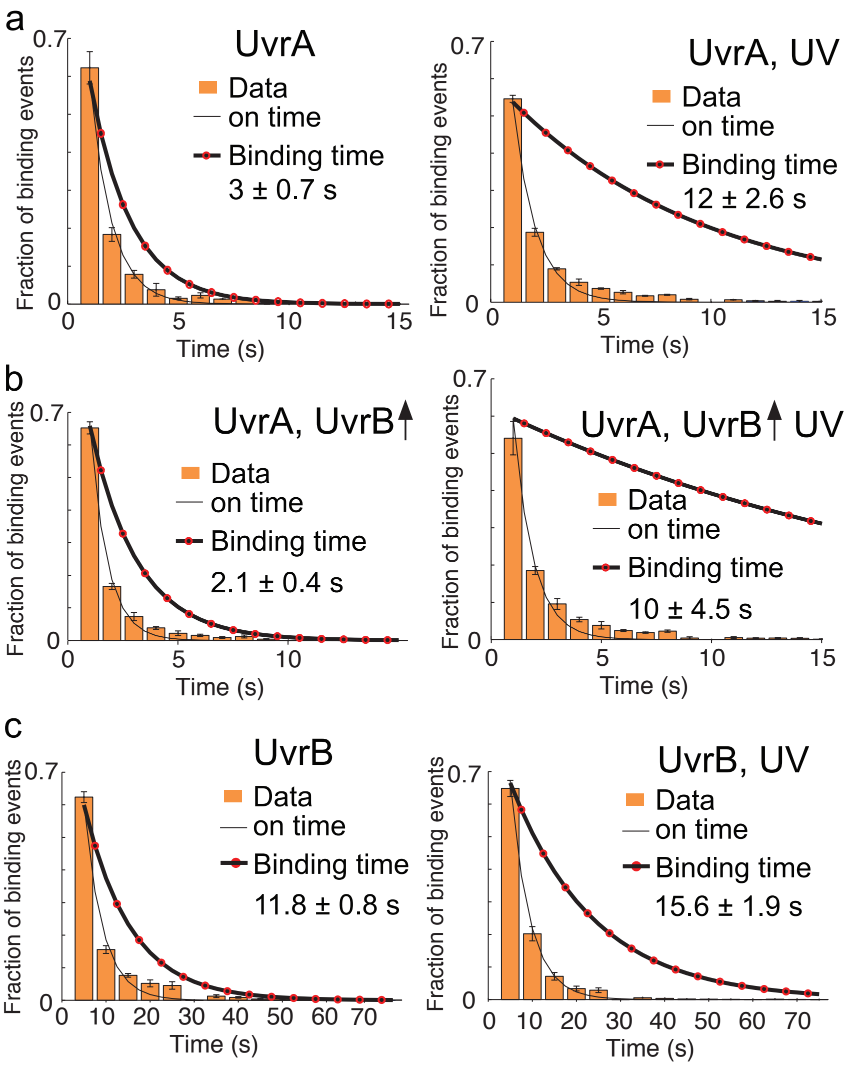

Supplementary Figure 3. Dwell times of UvrA and UvrB. a) On-time distributions for immobile UvrA-PAmCherry imaged with 1 s exposure times before (left) and after (right) UV exposure. Single exponential fits (solid lines) and photobleaching-corrected binding time distributions (dashed circled lines). Photobleaching times were measured by imaging cells with UvrA-PAmCherry fusion fixed with paraformaldehyde. Error bars shows S.E.M. of three experimental repeats. **b**) On-time distributions for immobile UvrA-PAmCherry after overexpression of unlabelled UvrB, imaged with 1 s exposure times. **c**) On-time distributions for immobile UvrB-PAmCherry imaged with 1 s exposure times followed by a 4 second delay. Immobile UvrB-PAmCherry recruited to non-damaged DNA was imaged in cells overexpressing unlabelled UvrA.

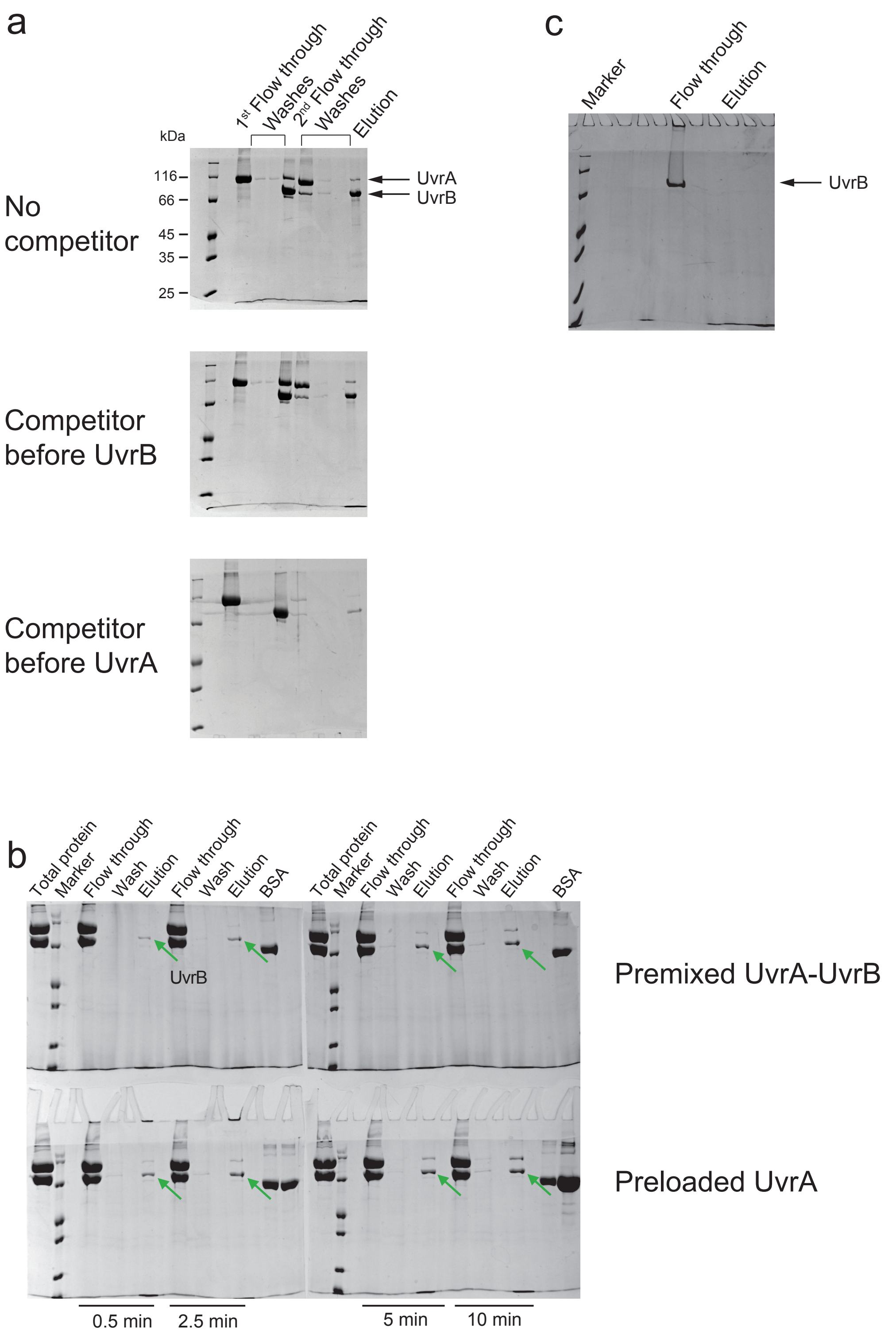

Supplementary Figure 4. *In vitro* UvrB loading assay. a) Example gels showing fractions from the competition experiments in all three conditions tested (for complete details see Methods). Pierce Unstained Protein MW Marker was used. **b**) Example gels from the UvrB recruitment time-course experiment. UvrB was either premixed with UvrA before addition to the damaged DAN, or added to damaged DNA

preloaded with UvrA. Green arrows indicate the position of UvrB. **c**) In the absence of UvrA, no UvrB was loaded onto the damaged DNA.

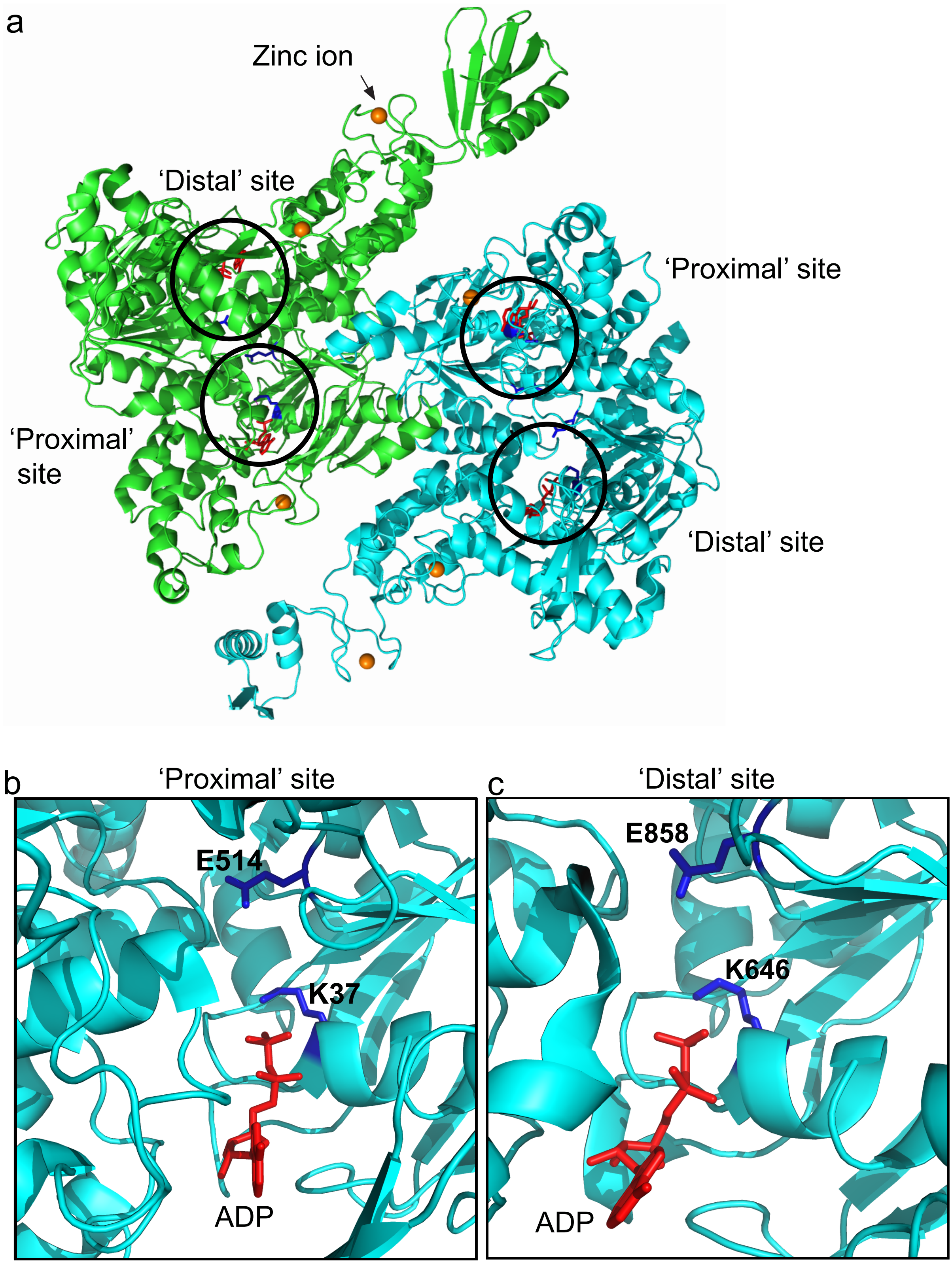

Supplementary Figure 5. Architecture of UvrA dimer. a) Structure of UvrA dimer from *Bacillus stearothermophilu* (pdb: 2R6F) with the 'proximal' and 'distal' ATPase sites highlighted. Numbering of amino acids shown is for UvrA from *E. coli*. b) Close up view of the proximal ATPase site. The K37 residue mutated to impair ATP binding, and the E514 residue mutated to impair ATP hydrolysis coloured in blue. c) Close up view of the distal binding site. The K646 residue mutated to impair ATP binding, and the E858 residue mutated to impair ATP hydrolysis coloured in blue.

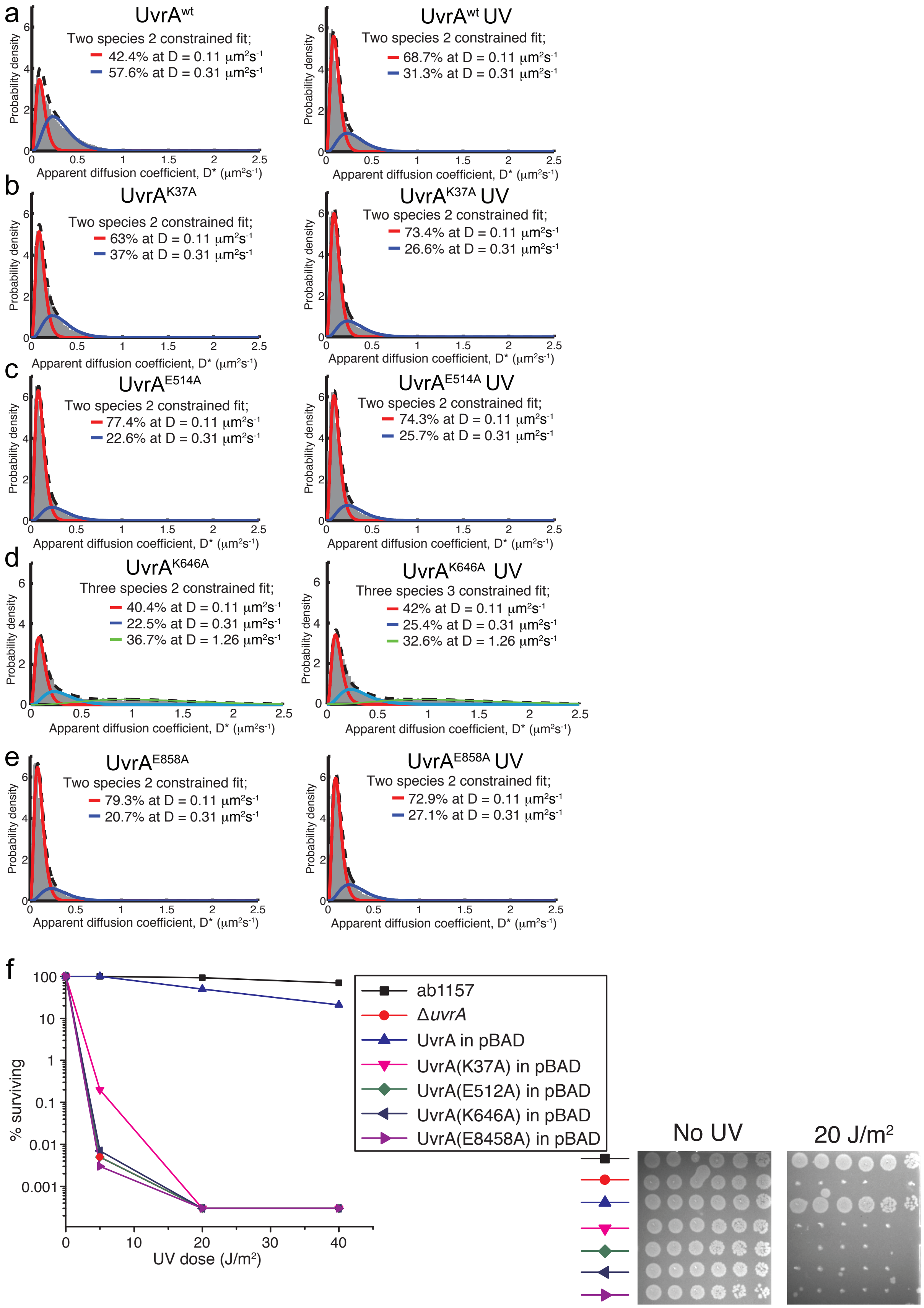

Supplementary Figure 6. Mobility and UV sensitivity of UvrA ATPase mutants. The distributions of apparent diffusion coefficients (*D**) for wt and ATPase mutants of

UvrA. All UvrA-PAmCherry variants were expressed from a pBAD plasmid at a low level (no arabinose added), in a strain with the endogenous *uvrA* and *mfd* genes deleted. **a**) The *D** distribution for wt UvrA-PAmCherry in cells not exposed to UV (left, 35846 trajectories) and after UV exposure (right, 25517 trajectories). The distribution was fitted with a two species model with both values constrained at D* values obtained for wt UvrA (Fig. 1b). **b**) The *D** distribution of $UvrA^{K37A}$-PAmCherry (proximal site, impaired ATP binding), with (16258 trajectories) and without (14195 trajectories) UV exposure. The distribution was fitted as above. **c**) The D* distribution of $UvrA^{E514A}$-PAmCherry (proximal site, inhibited ATP hydrolysis), with (12671 trajectories) and without (8872 trajectories) UV exposure. The distribution was fitted as above. **d**) The *D** distribution of $UvrA^{K646A}$-PAmCherry (distal site, impaired ATP binding), with (4075 trajectories) and without (11960 trajectories) UV exposure. The distribution could not be fitted with a two species model. Instead, three species model was used with two species constrained (D_{imm} = 0.11 $\mu m^2 s^{-1}$, D_{slow} = 0.31 $\mu m^2 s^{-1}$) and third one unconstrained. **e**) The *D** distribution of $UvrA^{KE858A}$-PAmCherry (distal site, impaired ATP hydrolysis), with (9805 trajectories) and without (8628 trajectories) UV exposure. **f**) UV sensitivity assay for UvrA ATPase mutants expressed at a low level from a pBAD plasmid (no arabinose added) in a strain with the endogenous *uvrA* gene deleted. Controls of wt AB1157, and Δ*uvrA* cells complemented with wt UvrA are also shown.

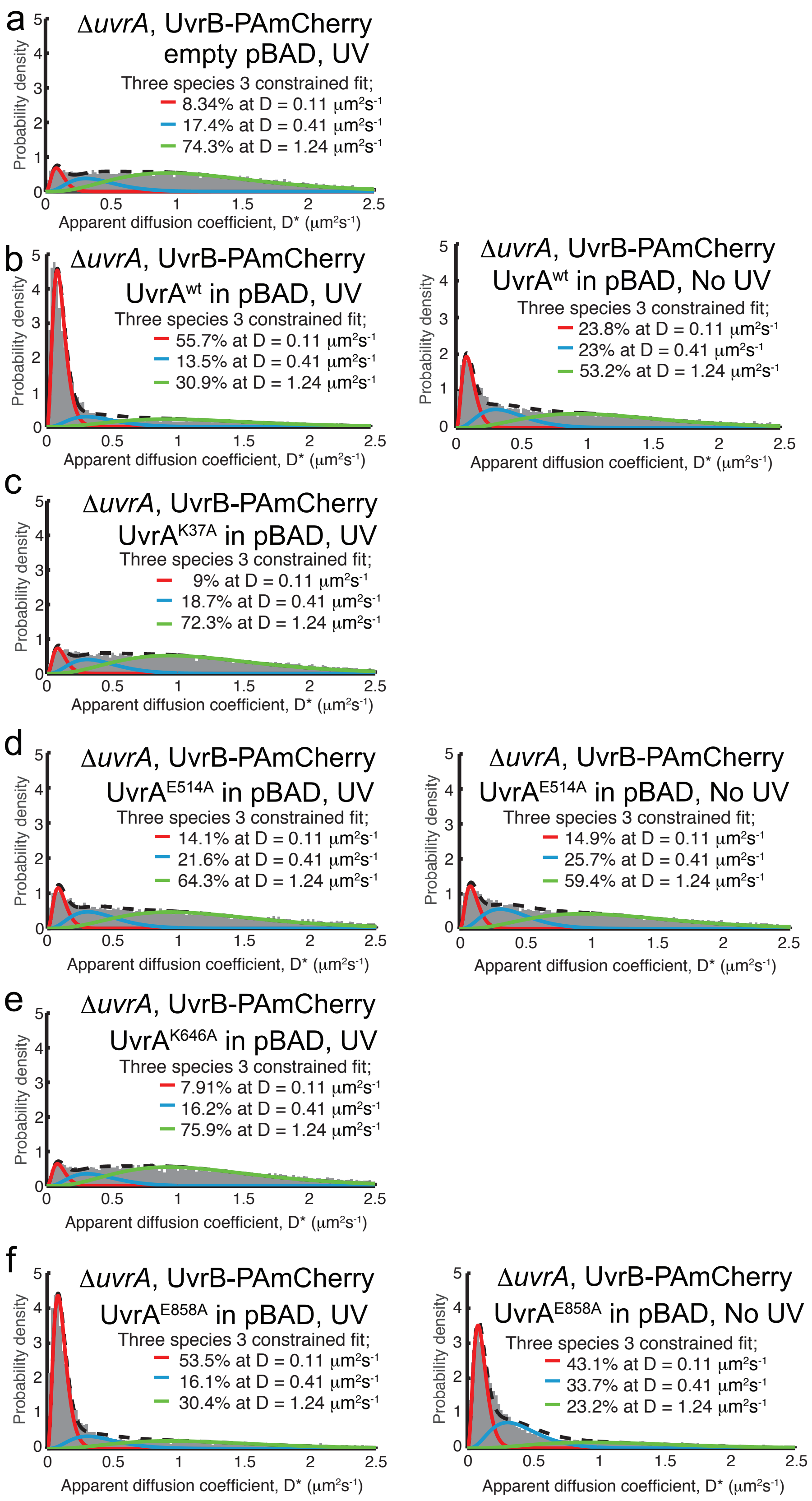

Supplementary Figure 7. Recruitment of UvrB by UvrA ATPase mutants. The distributions of apparent diffusion coefficients (*D*)* for endogenously expressed UvrB-PAmCherry, in Δ*uvrA* cells, complemented with wt UvrA or UvrA ATPase mutants

expressed at a low level (no arabinose added) from pBAD plasmid. Conditions after UV exposure are shown on the left and without UV exposure on the right. All distributions were fitted with three species model with all three *D** values constrained at values obtained for wt UvrB (Fig. 4a). **a**) An empty pBAD24 plasmid (14271 trajectories). **b**) A plasmid carrying wt UvrA (4942 trajectories after UV, and 7385 trajectories for no UV treatment). **c**) a plasmid carrying UvrA^{K37A} (proximal site, impaired ATP binding, 14768 trajectories). **d**) UvrA^{E514A} (proximal site, impaired ATP hydrolysis), 4211 trajectories after UV, and 5383 trajectories for no UV treatment. **e**) UvrA^{K646A} (distal site, impaired ATP binding, 13044 trajectories). **f**) UvrA^{KE858A} (distal site, impaired ATP hydrolysis), 5011 trajectories after UV treatment and 4002 trajectories for no UV treatment.

Strain	Genotype
AB1157	F$^-$, λ$^-$, rac$^-$, *thi-1, hisG4, Δ(gpt-proA)62, argE3, thr-1, leuB6, kdgK51, rfbD1, araC14, lacY1, galK2, xylA5, mtl-1, tsx-33, supE44(glnV44), rpsL31(strR), qsr'-0, mgl-51* [2]
PZ159	*uvrA:: PAmCherry kan*
PZ160	*uvrB::PAmCherry kan*
PZ161	Δ*uvrB kan*
PZ162	Δ*uvrA kan*
PZ168	*uvrA::PAmCherry kan;* Δ*uvrB frt*
PZ167	*uvrB::PAmCherry kan;* Δ*uvrA frt*
PZ187	*uvrA::PAmCherry frt;* Δ*mfd kan*
PZ198	*uvrA::PAmCherry frt; Δphotolyase kan*
PZ200	HUalpha::PAmCherry

Supplementary Table 1. Bacterial strains. All strains were in the AB1157 background and were constructed for this study. Abbreviations: *kan*, kanamycin resistance gene; *frt*, FLP site-specific recombination site.

Name	Description
UvrApamcherryfor	TCGCGGAGTGCGAAGCATCACACACGGCACGCTTCCTTAAGCCGATGCTGTCG GCT GGC TCC GCT GCT GGT TC
UvrApamcherryrev	GGTGCAACTCTGAAAGGAAAAGGCCGCTCAGAAAGCGGCCTTAACGATTAGAGGATCCCATATGAATATCCTCC
UvrBpamcherryfor	AAATTCGTGACCAGTTGCATCAGCTGCGTGAGCTGTTTATCGCGGCATCGTCG GCT GGC TCC GCT GCT GGT TC
UvrBpamcherryrev	CGCATCAGGCTGTTTTCCGTTTGTCATCAGTCTTCTTCGCTATCCTGTTAGAGGATCCCATATGAATATCCTCC
UvrAdeletionFor	ACCCATAATCTCAAAAACATCAACCTCGTTATCCCCCGCGACAAGCTCATGTAACCCGGGTGTAGGCTGGAG
UvrAdelefionRev	CTTCGCACTCCGCGACGGTTTCTGGCGTACCGGAGACGAGGATCTCGCCGGAATATCCTCCTTAGTTCC
UvrBdeletionFor	GCCAGAGGCGATTCGACGTCTCGAAGAGGGGCTGGAAGATGGCCTGGCGCGTAACCCGGGTGTAGGCTGGAG
UvrBdelefionRev	TTTGCGCCGCTTCTTCGAACTCCAGATTCTGCGCGTGTTGCATCATCAACGAATATCCTCCTTAGTTCC
MfdDeletionfor	TCTGCTGGGCGAGTTAACCGGCGCAGCCTGTGCAACGCTGGTAGCGGAAAGTAACCCGGGTGTAGGCTGGAG
MfdDeletionrev	TTAAGCGATCGCGTTCTCTTCCAGTTCACGCATAAACTGGCGTACCCATTGAATATCCTCCTTAGTTCC
Photolyasedelfor	ATGACTACCCATCTGGTCTGGTTTCGCCAGGATTTACGTCTGCACGATAAGTAACCCGGGTGTAGGCTGGAG
Photolyasedelrev	CTTCTTTGTGCTCGACTATCGGTTGCGGATAATCCAGCGTCACACCTGCTGAATATCCTCCTTAGTTCC
HUalphapamcherryfor	CTAACGTACCGGCATTTGTTTCTGGCAAGGCACTGAAAGACGCAGTTAAGTCGGCT GGC TCC GCT GCT GGT TC
HUalphapamcherryrev	AAAAGGGGTGAAACCACCCCTTCGTTAAAACTGTTCACTGCCACGCAATCTTAGAGGATCCCATATGAATATCCTCC
UvrApBADfor	ATATACCATGGATAAGATCGAAGTTCGGGGCGCCCG
UvrApBADrev	ATATATCTAGAGCCAGCCGACAGCATCGGCTTAAGGAAGCGTGCCGTGTG
UvrBpBADfor	GATTCGGTACCAATGAGTAAACCGTTCAAACTGAATTCC
UvrBpBADrev	GATTCAAGCTTTTACGATGCCGCGATAAACAGC
K37A FOR	CTTTCGGGTTCTGGCGCGTCCTCGCTCGCTTTC
K37A REV	GAAAGCGAGCGAGGACGCGCCAGAACCCGAAAG
K646A FOR	GGGTTTCAGGTTCCGGTGCGTCGACGCTGATTAACG
K646A REV	CGTTAATCAGCGTCGACGCACCGGAACCTGAAACCC
E514A FOR	GTTATGTACGTGCTGGACGCGCCGTCTATCG
E514A REV	CGATAGACGGCGCGTCCAGCACGTACATAAC
E858A FOR	TGTATATTCTCGACGCGCCGACCACCGGTC
E858A REV	CATATAAGAGCTGCGCGGCTGGTGGCCAGA
EcUvrA-pET28for	GAAGTCTACCAGGAACAAACCGGTGGATCCATGGATAAGATCGAAGTTCGGGG
EcUvrA-pET28rev	GATCTCAGTGGTGGTGGTGGTGGTGCTCGAGTTACAGCATCGGCTTAAGGAAGC
EcUvrB-pET28for	GAAGTCTACCAGGAACAAACCGGTGGATCCATGAGTAAACCGTTCAAACTGAA

EcUvrB-pET28rev	GATCTCAGTGGTGGTGGTGGTGGTGCTCGAGTTACGATGCCGCGATAAACAGCT
EcUvrA-pET28rev	GATCTCAGTGGTGGTGGTGGTGGTGCTCGAGTTACGATGCCGCGATAAACAGCT
MJ12-F26	GACTACGTACTGTTACGGCTCCATCFCTACCGCAATCAGGCCAGATCTGC<br>F- position where (5-C6-amino dT) was incorporated and labelled with fluorescein
MJ13	GCAGATCTGGCCTGATTGCGGTAGCGATGGAGCCGTAACAGTACGTAGTC
MJ13-biotin	Biotin-GCAGATCTGGCCTGATTGCGGTAGCGATGGAGCCGTAACAGTACGTAGTC
MJ12	GACTACGTACTGTTACGGCTCCATCTCTACCGCAATCAGGCCAGATCTGC

Supplementary Table 2. Oligonucleotides. Oligonucleotide sequences used for constructing flourescent fusions and overexpression plasmids. MJ oligonucleotides show sequences used for in vitro magnetic beads experiments.

Name	Description
pZ84	Full length UvrA cloned into pBAD24 using NcoI and HindIII cloning sites
pZ85	Full length UvrB cloned into pBAD24 using KpnI and HindIII cloning sites
pZ86	UvrA-PAmCherry cloned into pBAD24 using NcoI and HindIII cloning sites
pZ88	$UvrA^{E514A}$-PAmCherry cloned into pBAD24 using NcoI and HindIII cloning sites
pZ89	$UvrA^{K646A}$-PAmCherry cloned into pBAD24 using NcoI and HindIII cloning sites
pZ90	$UvrA^{E858A}$-PAmCherry cloned into pBAD24 using NcoI and HindIII cloning sites
pZ92	$UvrA^{K37A}$ cloned into pBAD24 using NcoI and HindIII cloning sites
pZ93	$UvrA^{E514A}$ cloned into pBAD24 using NcoI and HindIII cloning sites
pZ94	$UvrA^{K646A}$ cloned into pBAD24 using NcoI and HindIII cloning sites
pZ95	$UvrA^{E858A}$ cloned into pBAD24 using NcoI and HindIII cloning sites
pET28-UvrA	Full length UvrA cloned into pET28 using BamHI i XhoI
pET28-UvrB	Full length UvrB cloned into pET28 using BamHI i XhoI

Supplementary Table 3. Plasmids. Plasmids for expression of unlabelled and PAmCherry fluorescent fusions of UvrA, UvrB, and UvrA ATPase mutants.

Supplementary Methods

1. Bacterial strains

All strains were derivatives of *Escherichia coli* K-12 AB1157[2] and are listed in Supplementary Table 1. The plasmids and oligonucleotides used are shown in

Supplementary Table 2. Replacement of genes with C-terminal fluorescent fusions was performed using λ-Red recombination[3] and fusion genes were moved into the final strain by P1 phage transduction[4]. The flexible 11 aminoacid linker (SAGSAAGSGEF) was introduced between full length of each protein and PAmCherry protein. All deletions were constracted *de novo*. The first and last 50bp of coding sequence was left in order to prevent removal of potential regulatory elements for other genes. For multiple insertions of modified genes, the *kan^r* gene was removed using site-specific recombination through expression of the Flp recombinase from plasmid pCP20[3]. Correct insertion of the fragment into the chromosome was evaluated by PCR using primers flanking the insertion site. Strains carrying UvrA or UvrB PAmCherry fusions expressed from their endogenous promoter showed no increase in the sensitivity to UV compare to AB1157 wild-type (Supplementary Figure 1a). Exposure to UV light resulted in an increase of immobile molecules for both UvrA-PAmCherry and UvrB-PAmCherry as a result of active repair, further confirming the functionality of the fusion proteins.

UvrA mutants with impaired ATP binding were constructed based on previously published work[5,6]. Each UvrA monomer contains two ABC ATPase structural modules. To prepare variants of UvrA with altered ATP hydrolysis, conserved glutamates from Walker B motifs were substituted with alanine. Mutational analysis of another protein in the ABC transporter superfamily, MJ0796, has shown that replacement of this residue leads to a protein which cannot efficiently hydrolyze ATP[7]. Additionally, our own UvrA crystal structure[8] and a structure published by others[9], together with our unpublished results, suggested that the E514 and E858 residues are in a position to act as the catalytic carboxylate. Plasmids were constructed using conventional cloning techniques. PBAD constructs were cloned with the indicated restriction enzymes and natural codon stops were used. Expression of Both UvrA and UvrB constructs was controlled by the pARA promoter; pZ84 produced a full length UvrA protein identical to the wt protein, pZ85 produced a UvrB protein extended at N-terminus by three aminoacids (MVP) as a consequence of cloning site selection. Single amino acid substitutions were introduced using QuikChange method (Agilient Technologies); for oligonucleotides used see Supplementary Table 2.

2a. UV survival assay

Cells were grown in LB media to A_{600} ~0.6. Serial dilutions up to 10^{-7} were prepared and 10 µl of each dilution was placed on LB plates in duplicate. One plate was irradiated with 254 nm UV light (Stratagene, UV Stratalinker 1800) and the other plate was use as a control of growth without exposure to UV. The number of colonies obtained after 20h incubation at 37°C was recorded and the percent surviving was calculated from the plating efficiency of the non-irradiated control. At least three independent experiments were performed for each strain. This was repeated for four different UV doses; 0 J/m^2, 5 J/m^2, 20 J/m^2, 40 J/m^2, and the mean percentage survival was plotted as a function of UV dose. It was previously estimated that UV exposure (254 nm) introduces ~40 lesions/chromosome per 1J/m^2 [10].

2b. Cell preparation.

Strains were streaked onto LB plates with appropriate antibiotics. Single colonies were inoculated into M9 glycerol (0.2%) and grown overnight at 37ºC to A_{600} 0.4-0.6, then diluted into fresh M9 and grown to A_{600} 0.1. Cells were centrifuged and immobilized on agarose pads between two glass coverslips (0.17mm thickness, heated to 500ºC for 1 h to remove any fluorescent background particles). We prepared 1% agarose pads by mixing low-fluorescence 2% agarose (Bio-Rad) in dH_2O 1:1 with 2x growth medium.

Where indicated, prepared cells immobilized on agarose pads were exposed with 50 J/m^2 of 254 nm UV light (Stratagene, UV Stratalinker 1800) 5 minutes prior to imaging. Cells were imaged between 5 and 15 minutes after the exposure. For overexpression experiments, unlabelled UvrA or UvrB was expressed from a pBAD plasmid[11], by inducing with 0.2% arabinose for 1 h prior to imaging. For experiments imaging with UvrA ATPase mutants (Figure 6b, Supplementary Fig. 6) the mutant UvrA-PAmCherry fusions were expressed from a pBAD plasmid with no induction (leaky expression only) in a strain with the endogenous *uvrA* and *mfd* genes deleted. For experiments imaging UvrB-PAmcherry recruitment to DNA in the presence of UvrA mutants (Figure 6c, Supplementary Fig. 7), unlabelled UvrA mutants were expressed from a pBAD plasmid with no induction, and 0.2% glucose added to further supress leaky expression, in a strain with the endogenous *uvrA* gene deleted, and an endogenous replacement of the *uvrB* gene with the *uvrB-PAmCherry* fusion.

For photobleaching controls using fixed cells, centrifuged cells prepared as above were resuspended into 2.5% paraformaldehyde in M9 media and fixed for 45 min shaking at 22ºC. Fixed cells were washed, then immobilized on agarose pads as for live cells.

3. PALM microscopy

Live cell single-molecule-tracking PALM was performed on a custom-built total internal reflection fluorescence (TIRF) microscope built around the Rapid Automated Modular Microscope (RAMM) System (ASI Imaging). Photoactivatable mCherry activation was controlled by a 405 nm laser and excitation with 561 nm. All lasers were provided by a multi-laser engine (iChrome MLE, Toptica). At the fibre output, the laser beams were collimated and focused (100x oil immersion objective, NA 1.4, Olympus) onto the sample under an angle allowing for highly inclined thin illumination[12]. Fluorescence emission was filtered by a dichroic mirror and notch filter (ZT405/488/561rpc & ZET405/488/561NF, Chroma). PAmCherry emission was projected onto an EMCCD camera (iXon Ultra, 512x512 pixels, Andor). The pixel size was 96 nm. Transmission illumination was provided by an LED source and condenser (ASI Imaging). Sample position and focus were controlled with a motorized piezo stage, a z-motor objective mount, and autofocus system (MS-2000, PZ-2000FT, CRISP, ASI Imaging).

4. Localization and tracking.

PALM data for single-molecule-tracking analysis was localized using custom-written MATLAB software (MathWorks). Fluorophore images were identified for localisation by band-pass filtering and applying an intensity threshold to each frame of a super-resolution movie. Candidate positions were used as initial guesses in a two-dimensional elliptical Gaussian fit for high-precision localisation. Free fit parameters were x-position, y-position, x-width, y-width, elliptical rotation angle, intensity, background. Single-particle tracking analysis was performed by adapting the MATLAB implementation of the algorithm described in ref[13]. Positions were linked to a track if they appeared in consecutive frames within a window of 5 pixels (0.48 µm) for UvrA and 7 pixels for UvrB (0.67 µm). In rare cases when multiple localizations fell within the tracking radius, tracks were linked such that the sum of step distances was minimized. We used a 'memory' parameter of 1 frame to allow for transient (1

frame) disappearance of the fluorophore image within a track due to blinking or missed localisation.

5. Molecule counting

We counted the total number of UvrA or UvrB molecules by recording long movies (31000 frames), until no further activation was observed. Cells were segmented from transmission images using MicrobeTracker[14]. Localisations within cell boundaries were tracked and the number of tracked molecules per cell established. We note that the copy numbers presented here may be underestimates of the true copy numbers, since only 49% of PAmCherry were shown to be photoactivatable in studies in eukaryotic cells[15]. However, the ratio between copy numbers of UvrA and UvrB is not affected by this underestimation. We find that both are expressed at similar levels with, on average, ~85 copies each per cell. This resolves contradictory earlier studies, which have reported both more UvrA than UvrB molecules[16], and more UvrB than UvrA molecules present in cells[17].

6. Measuring the diffusion of PAmCherry labeled proteins.

We determined the mobility of each molecule by calculating an apparent (or nominal) diffusion coefficient, *D**, from the one-step mean-squared displacement (MSD) of the track using:

$$D^* = \frac{1}{4n\Delta t}\sum_{i=1}^{n}[x(i\Delta t) - x(i\Delta t + \Delta t)]^2 + [y(i\Delta t) - y(i\Delta t + \Delta t)]^2$$

Where $x(t)$ and $y(t)$ are the coordinates of the molecule at time t, the frame time of the camera is Δt, and n is the number of steps in the trajectory. Tracks shorter than $n = 4$ steps long were discarded for this analysis because the higher uncertainty in *D** value.

For a molecule with apparent diffusion coefficient *D*, the probability distribution of obtaining a single-molecule *D** value, x, is given by:

$$f(x; D, n) = \frac{({}^{n}/_{D})^{n} x^{n-1} e^{-nx}/_{D}}{(n-1)!}$$

Where n is the number of steps in the trajectory. In order to determine the apparent diffusion coefficient, *D*, from the population of individual single-molecule *D** values, longer tracks were truncated after 5th localization (i.e. $n = 4$). The *D** distribution, x, was then fitted to the $n = 4$ analytical expressionequation:

$$f(x; D) = \frac{({}^{4}/_{D})^{4} x^{3} e^{-4x}/_{D}}{6}$$

Fits were performed using maximum likelihood estimation in MATLAB, and errors were estimated as the SD in each estimated parameter using bootstrap resampling with 100 resamples, rounded up to the nearest 0.01 $\mu m^2 s^{-1}$. A single species model fits poorly to the data (Supplementary Fig. 1d). We reasoned that at least two species with different mobilities are present: mobile molecules diffusing and binding only transiently to DNA, and immboile molecules bound to DNA for the entire trajectory. We therefore introduced a second species:

$$f(x; D_1, D_2, A) = \frac{A({}^{4}/_{D_1})^{4} x^{3} e^{-4x}/_{D_1}}{6} + \frac{(1-A)({}^{4}/_{D_2})^{4} x^{3} e^{-4x}/_{D_2}}{6}$$

Where D_1 and D_2 are the diffusion coefficients of the two different species, and A and $1 - A$ are the fraction of molecules found in each state. For fitting to UvrB, a third species was added in a similar fashion.

The localisation uncertainty in each measurement, σ_{loc} , manifests itself as a positive offset in the *D** value of $\sigma_{loc}^2/\Delta t$[18]. Based on the estimated localisation uncertainty of ~40 nm for our measurements, we expected a positive shift in the mean *D** value of immobile molecules to ~0.1 $\mu m^2 s^{-1}$. We verified this in previous work by using a well charaterized control protein, DNA polymerase 1 (Pol1), which shows clearly distinct *D** populations for molecules specifically bound to DNA and those mobile molecules diffusing through the nucleoid searching for substrate[19]. Fitting the distribution of Pol1 D* values, imaged with the same aquisisiton settings on the same experimenetal setup, with a two diffusing species allowed us to

determine the D value of specifically bound molecules as D^* = 0.11 μm^{2}s^{-1} [1]. The apparent diffusion coefficient for immobile molecules was consistent for UvrA and UvrB presented here, but also with our previous results imaging other *E. coli* DNA-binding proteins using the same experimental setup[1,20]. Using this D^* value for bound molecules to constrain one D^* species, and allowing a second unconstrained D^* species fits well to the data giving two populations; immobile molecules, D_{imm} = 0.11 μm^{2}s^{-1}, and molecules diffusing slowly, D_{slow} = 0.31 μm^{2}s^{-1} (Fig.2a). Subsequently, except where indicated, we fit all UvrA distributions with two species constrained at D values; D_{imm} =0.11 μm^{2}s^{-1}, D_{slow} =0.31 μm^{2}s^{-1}. We note that allowing two unconstrained fits to the UvrA distribution gave the same results.

UvrB D^* distribution was much broader than that of UvrA, and fitted poorly to a two species model (Supplementary Figure 1f). We reasoned that third population with faster diffusion exists. Fiting three diffusing species to UvrB data with immobile population constrained at D_{imm} = 0.11μm^{2}s^{-1}, resulted in a good fit to the data, with D_{slow} = 0.41 μm^{2}s^{-1}, and D_{fast} = 1.24 μm^{2}s^{-1} (Fig. 4a). Subsequently, we fit all UvrB distributions with three species constrained at D values; D_{imm} = 0.11 μm^{2}s^{-1}, D_{slow} = 0.41 μm^{2}s^{-1} and D_{fast} = 1.24 μm^{2}s^{-1}.

7. Simulating protein diffusion

Estimates of the diffusion coefficient of unconjugated fluorescent proteins diffusing in *E. coli* range between 7 μm^2/s (eYFP[21]), 8 μm^2/s (Venus[22]) and 10 μm^2/s (Dendra2[23]). The diffusion coefficient is inversely proportional to the Stokes radius, which we can roughly approximate from the relative sizes of the proteins (PAmCherry = 29 kDa; UvrA-PAmCherry dimers = 270 kDa). We would therefore expect free UvrA-PAmCherry to diffuse within the cytoplasm with D between 3.5 and 4.7 μm^{2}s^{-1}. For UvrB-PAmCherry monomers (105 kDa), this is between 4.9 and 6.5 μm^{2}s^{-1}.

However, the apparent diffusion observed experimentally through particle tracking does not take into account confinement due to the small size of bacteria, and other effects such as localisation error[19,24,25]. To determine the distribution of apparent diffusion coefficents we would expect to observe from our experiments, we simulated Brownian motion confined within a volume corresponding to the average size of cells imaged in experiments, defined as a cylindrical volume of length 2 μm long and 0.9 μm wide with hemispherical endcaps with a radius of 0.9 μm[19]. Each 15 ms frame was split into 300 sub-frames with Gaussian distributed displacements in

each sub-frame. Each molecule trajectory was given a random starting time to mimic stochastic photoactivation. The trajectory was then simulated with a duration sampled from an exponential distribution with a mean time equal to our experimentally determined photobleaching lifetime (~70 ms). The sub-frame distributions were then averaged to give a position for each frame, and a localization error sampled from a Guassian distribution with σ_{loc} = 40 nm was added. The list of simulated localizations, with their corresponding frame number was then analyzed using the same tracking algorithm with the same settings as used for the experimental data. The outputted tracks could then be analyzed in exactly the same way as experimental data. Molecules simulated with a D of 4 $\mu m^2 s^{-1}$ gave a mean D* value of 1.2 $\mu m^2 s^{-1}$. On the other hand, simulating immobile molecules (D = 0 $\mu m^2 s^{-1}$), gave a mean *D** of 0.11 $\mu m^2 s^{-1}$ as expected from the uncertainty in each localization. The expected distributons of apparent diffusion coefficients are shown in Fig. 1b.

We hypothesized that the slow diffusion, D_{slow} = 0.31 $\mu m^2 s^{-1}$, observed for mobile UvrA molecules was due to molecules undergoing transient interactions with DNA and therefore rapidly interconverting between the D_{free} and D_{imm} states. We simulated molecules which could rapidly interconvert the two states; D = 4 $\mu m^2 s^{-1}$ and D = 0 $\mu m^2 s^{-1}$. Simulated interconverting molecules with a transient binding was randomly sampled from an exponential with a mean of 0.08 ms, and a rebinding time sampled from an exponential with a mean of 0.92 ms shows that the experimentally observed D_{slow} = 0.31 $\mu m^2 s^{-1}$ can be recapitulated by a molecule rapidly interconverting between D = 4 $\mu m^2 s^{-1}$ and D = 0 $\mu m^2 s^{-1}$ (Fig. 1b).

8. Measuring long-lasting binding events

PALM movies to measure long duration binding events were recorded at low continuous 561-nm excitation intensities using 1 s exposure times[19,20]. At this exposure times mobile UvrA-PAmCherry molecules are motion blurred over a large fraction of the cell, whereas immobile UvrA-PAmCherry molecules still appear as point sources, producing a diffraction limited spot. Elliptical Gaussian fitting was used as described in section 6. Bound and mobile molecules were distinguished by the width of the elliptical fits, with thresholds short axis-width < 160 nm and long axis-width < 200 nm to identify bound molecules. The probability of observing a particular on-time is the product of the underlying binding-time probability and the bleaching probability. The bleaching-time distributions were measured independently using

UvrA-PAmCherry in cells fixed with paraformaldehyde, with the same acquisition and excitation conditions. On-time and bleaching-time distributions were fitted with single-exponential functions to extract exponential-time constants t_{on} and t_{bleach}, and the binding-time constant was calculated by $t_{bound} = t_{on} \cdot t_{bleach} / (t_{bleach} - t_{on})$. Stochastic photoactivation of UvrA-PAmCherry and UvrB-PAmCherry molecules before or during binding events does not influence our measurement, because the observed binding times follow an exponential distribution and are therefore memoryless.

UvrA was analysed with a 1s exposure time, however, UvrB in UV exposed cells had binding times similar to those observed for the bleaching control, indicating a longer time constant of the off event (thereafter refer to as dwell time). In order to image these longer binding times, we therefore imaged UvrB using a 4 s delay between each 1 s exposures. To determine the binding time of UvrB when recruited to DNA by UvrA when no damage is present, we used overexpression of unlabelled UvrA to induce recruitment of UvrB to non-damage sites. We conclude that this binding time represents UvrB loading and damage verification, but not repair. Measuring of the binding time for UvrB in wt cells was not performed, since we do not know if the small fraction of UvrA-dependent immobile UvrB molecules (4%, Fig. 4b) represents a basal level of DNA repair or false-positive recruitment of UvrB to non-damage DNA.

9. Protein purification

The *uvrA* and *uvrB* genes were amplified in a PCR reaction (see Supplementary Table 2 for appropriate oligonucleotides used) from *Escherichia coli* (K-12) genomic DNA and cloned into a pET28 expression vector (Novagen) using BamHI i XhoI restriction sites. Both proteins were expressed as N-terminal His-SUMO (6xHistidine – Small Ubiquitin-like Modifier) fusion proteins. UvrA was overexpressed in BL21(DE3) STAR^{TM} strain (Invitrogen) overnight at 18°C after induction with 0.4 mM IPTG. UvrB was overexpressed in BL21(DE3) STAR^{TM} strain (Invitrogen) in Super Broth Base including trace elements autoinduction media (FORMEDIUM). Bacterial cells were suspended in 40 mM NaH2PO4 (pH 7.0) buffer with 5% (v/v) glycerol, 150 mM NaCl and 10 mM β-mercaptoethanol (buffer A) with lysozyme at a concentration of 1 mg/ml, and a mix of protease inhibitors. After 30 minutes incubation on ice, the salt concentration was increased to 1 M and suspensions were sonicated. Lysates

were cleared by ultracentrifugation and proteins were purified using nickel affinity chromatography. Before elution, the resin was washed with the buffer A containing 120 mM imidazole and 1 M NaCl. Eluted fusion proteins were next digested with Ulp1 protease (during overnight dialysis to buffer A with 1 M NaCl and 40 mM imidazole, which resulted in full length proteins identical to wild type) and again purified using nickel affinity chromatography. The final purification step was done on a gel filtration column (HiLoad 16/60 Superdex 200 prep grade) equilibrated with 20 mM Hepes (pH 7.0) buffer with 5% glycerol, 1 M NaCl, 1 mM DTT, and 0.5 mM EDTA. Pure proteins were stored at 4°C.

10. *In vitro* UvrB loading assay

The UvrB competition assay was designed to test if UvrA bound to damaged DNA, could load UvrB onto DNA lesions directly from solution. To this end we preloaded UvrA onto biotinylated DNA containing a fluorescein lesion (MJ12-F26-biotin; see Supplementary Table 2 for the sequences of oligonucleotides used) and subsequently bound this complex to streptavidin-coated beads. We tested whether addition of 50 bp competitor DNA containing the same fluorescein lesion (MJ12-F26), affected the loading of UvrB onto damaged DNA.

The assay was performed as follows: 485 μl of 50 mM Tris (pH 7.5) buffer containing 75 mM KCl, 10 mM $MgCl_2$, 0.1% (v/v) Tween-20 and 1 mM DTT (buffer X) with 1 mM ATP was mixed with 5 μl of biotinylated damaged DNA (final concentration - 60 nM). Next, 5 μl of UvrA protein (diluted with buffer X containing 1 mM ATP) was added (final dimer concentration - 60 nM) and the reaction was incubated at 37°C for 60 seconds with mixing. Then 5 μl of beads (500 μg Dynabeads® MyOne™ Streptavidin T1) suspended in buffer X with 1 mM ATP was added and the reaction was further incubated at 37°C for 90 seconds. Beads were pelleted with a magnet and washed three times with buffer X. Beads were resuspended in 495 ml of buffer X with 1 mM ATP and 5 μl of UvrB protein (diluted with buffer X containing 1 mM ATP) was added (final concentration - 120 nM). The reaction was incubated at 37°C for 150 seconds with mixing. The beads were pelleted with a magnet and washed three times with buffer X containing 1 M NaCl and subsequently re-suspended in 10 μl of H_2O and 5 μl of SDS-PAGE loading buffer. 5 μl of the competitor DNA (final concentration - 600 nM) was added either before the addition of UvrB, or together

with the biotinylated DNA. As a control for the intermediate steps of the reaction, 400 µl of each fraction (flow-through or wash) was mixed with 1600 µl of cold (-20°C) acetone, and incubated overnight at -20°C. The next day, the fractions were centrifuged at 20000×g for 15 minutes at 4°C, and the supernatants were discarded. The dried protein pellets were re-suspended in 10 µl of H_2O and 5 µl of SDS-PAGE loading buffer. All fractions, including beads, were mixed with SDS-PAGE loading buffer, incubated for 2-3 minutes at 95°C, and resolved on 9% polyacrylamide SDS-PAGE gels. Gels were fixed and stained with Bio-Safe Coomassie G-250 Stain (Bio-Rad).

The time-course experiment was performed with the same final concentrations of UvrA, UvrB, and biotynilated damaged DNA as for the competition assay. In addition, an excess of undamaged nonbiotinylated DNA (600nM) was added to mimic the conditions *in vivo*. Loading of UvrB onto the damaged DNA was investigated in two reactions. In reaction A, UvrA and UvrB proteins were added together. In reaction B, UvrA was incubated with biotinylated DNA prior the addition of UvrB. The reaction was performed as follows: buffer X containing 1 mM ATP was mixed with: 20 µl of biotinylated damaged DNA and 20 µl of undamaged DNA (reaction A) or 20 µl of UvrA protein and 20 µl of biotinylated damaged DNA (reaction B). Next, reactions were incubated for 150 seconds at 37°C with mixing. 20 µl of beads (2 mg Dynabeads® MyOne™ Streptavidin T1) suspended in buffer X with 1 mM ATP were added and the reactions were further incubated with mixing at 37°C for 30 seconds. Next 20 µl of UvrA and 20 µl of UvrB, premixed and incubated for 60s at 37°C were added to reaction A and 20 µl of nondamaged DNA and 20 µl of UvrB were added to reaction B. Reactions were incubated at 37°C with mixing and 500 µl samples were taken after 30 seconds, 2.5, 5 and 10 minutes. Loading reactions were stopped by the increase of NaCl concentration to 1 M. Beads were pelleted with a magnet and washed three times with 500 µl of buffer X with 1 M NaCl. Beads were re-suspended in 10 µl of H2O and 5 µl of SDS-PAGE loading buffer. 0.8 volume of beads supernatant or wash fractions were precipitated with ice cold (-20°C) acetone and incubated for 1 hour at -20°C. Next fractions were centrifuged at 20000×g for 15 minutes at 4°C, and the supernatants were discarded. The dried protein pellets were re-suspended in 10 µl of H2O and 5 µl of SDS-PAGE loading buffer. All fractions, including beads, were incubated for 2-3 minutes at 95°C and resolved quantitatively

on 9% polyacrylamide SDS-PAGE gels. Gels were fixed and stained with Bio-Safe Coomassie G-250 Stain (Bio-Rad).

11. Intracelular spatial distribution of UvrA and UvrB molecules

Analysis was performed in Matlab (Mathworks). Cells were segmented from brightfield images using MicrobeTracker, giving a cell outline and a cell midline, and the positions of molecule localisations were determined relative to the cell midline, with the *x* axis defined as the cell short axis and the *y* axis defined as the cell long axis. The x any y posistions of each loaclisations was normalised relative to the cell width and length, respectively (Supplementary Fig. 1e). The 2D histograms of the distributions of localisations shown were generated by binning cells into different lengths (2-3 μm and 3.5-4.5 μm long), with short cells having a single nucleoid located in the center of the y axis, and long cells having two nucleoids. The average positions of nucloid regions was verified by performing the same analysis with the nonspecific nucleoid associated protein, HU, which was labelled with PAmCherry and imaged and tracked using the same protocol as for UvrA and UvrB.

Supplementary References

1 Stracy, M. *et al.* Live-cell superresolution microscopy reveals the organization of RNA polymerase in the bacterial nucleoid. *Proceedings of the National Academy of Sciences of the United States of America*, doi:10.1073/pnas.1507592112 (2015).

2 Bachmann, B. J. Pedigrees of some mutant strains of Escherichia coli K-12. *Bacteriological reviews* **36**, 525-557 (1972).

3 Datsenko, K. A. & Wanner, B. L. One-step inactivation of chromosomal genes in Escherichia coli K-12 using PCR products. *Proceedings of the National Academy of Sciences of the United States of America* **97**, 6640-6645, doi:10.1073/pnas.120163297 (2000).

4 Thomason, L. C., Costantino, N. & Court, D. L. E. coli genome manipulation by P1 transduction. *Current protocols in molecular biology / edited by Frederick M. Ausubel ... [et al.]* **Chapter 1**, Unit 1 17, doi:10.1002/0471142727.mb0117s79 (2007).

5 Myles, G. M., Hearst, J. E. & Sancar, A. Site-specific mutagenesis of conserved residues within Walker A and B sequences of Escherichia coli UvrA protein. *Biochemistry* **30**, 3824-3834 (1991).

6 Thiagalingam, S. & Grossman, L. Both ATPase sites of Escherichia coli UvrA have functional roles in nucleotide excision repair. *The Journal of biological chemistry* **266**, 11395-11403 (1991).

7 Moody, J. E., Millen, L., Binns, D., Hunt, J. F. & Thomas, P. J. Cooperative, ATP-dependent association of the nucleotide binding cassettes during the catalytic cycle of ATP-binding cassette transporters. *The Journal of biological chemistry* **277**, 21111-21114, doi:10.1074/jbc.C200228200 (2002).

8 Jaciuk, M., Nowak, E., Skowronek, K., Tanska, A. & Nowotny, M. Structure of UvrA nucleotide excision repair protein in complex with modified DNA. *Nature structural & molecular biology* **18**, 191-197, doi:10.1038/nsmb.1973 (2011).

9 Pakotiprapha, D., Samuels, M., Shen, K., Hu, J. H. & Jeruzalmi, D. Structure and mechanism of the UvrA-UvrB DNA damage sensor. *Nature structural & molecular biology* **19**, 291-298, doi:10.1038/nsmb.2240 (2012).

10 Rudolph, C. J., Upton, A. L. & Lloyd, R. G. Replication fork stalling and cell cycle arrest in UV-irradiated Escherichia coli. *Genes & development* **21**, 668-681, doi:10.1101/gad.417607 (2007).

11 Guzman, L. M., Belin, D., Carson, M. J. & Beckwith, J. Tight regulation, modulation, and high-level expression by vectors containing the arabinose PBAD promoter. *Journal of bacteriology* **177**, 4121-4130 (1995).

12 Tokunaga, M., Imamoto, N. & Sakata-Sogawa, K. Highly inclined thin illumination enables clear single-molecule imaging in cells. *Nature methods* **5**, 159-161, doi:10.1038/nmeth1171 (2008).

13 Crocker, J. C. & Grier, D. G. When Like Charges Attract: The Effects of Geometrical Confinement on Long-Range Colloidal Interactions. *Physical review letters* **77**, 1897-1900, doi:10.1103/PhysRevLett.77.1897 (1996).

14 Sliusarenko, O., Heinritz, J., Emonet, T. & Jacobs-Wagner, C. High-throughput, subpixel precision analysis of bacterial morphogenesis and intracellular spatio-temporal dynamics. *Molecular microbiology* **80**, 612-627, doi:10.1111/j.1365-2958.2011.07579.x (2011).

15 Durisic, N., Laparra-Cuervo, L., Sandoval-Alvarez, A., Borbely, J. S. & Lakadamyali, M. Single-molecule evaluation of fluorescent protein photoactivation efficiency using an in vivo nanotemplate. *Nature methods* **11**, 156-162, doi:10.1038/nmeth.2784 (2014).

16 Lin, C. G., Kovalsky, O. & Grossman, L. DNA damage-dependent recruitment of nucleotide excision repair and transcription proteins to Escherichia coli inner membranes. *Nucleic acids research* **25**, 3151-3158 (1997).

17 Truglio, J. J., Croteau, D. L., Van Houten, B. & Kisker, C. Prokaryotic nucleotide excision repair: the UvrABC system. *Chemical reviews* **106**, 233-252, doi:10.1021/cr040471u (2006).

18 Michalet, X. & Berglund, A. J. Optimal diffusion coefficient estimation in single-particle tracking. *Physical review. E, Statistical, nonlinear, and soft matter physics* **85**, 061916 (2012).

19 Uphoff, S., Reyes-Lamothe, R., Garza de Leon, F., Sherratt, D. J. & Kapanidis, A. N. Single-molecule DNA repair in live bacteria. *Proceedings of the National Academy of Sciences of the United States of America* **110**, 8063-8068, doi:10.1073/pnas.1301804110 (2013).

20 Zawadzki, P. *et al.* The Localization and Action of Topoisomerase IV in Escherichia coli Chromosome Segregation Is Coordinated by the SMC Complex, MukBEF. *Cell Rep* **13**, 2587-2596, doi:10.1016/j.celrep.2015.11.034 (2015).

21 Kumar, M., Mommer, M. S. & Sourjik, V. Mobility of cytoplasmic, membrane, and DNA-binding proteins in Escherichia coli. *Biophysical journal* **98**, 552-559, doi:10.1016/j.bpj.2009.11.002 (2010).

22 Elf, J., Li, G. W. & Xie, X. S. Probing transcription factor dynamics at the single-molecule level in a living cell. *Science* **316**, 1191-1194, doi:10.1126/science.1141967 (2007).

23 Sanamrad, A. *et al.* Single-particle tracking reveals that free ribosomal subunits are not excluded from the Escherichia coli nucleoid. *Proceedings of the National Academy of Sciences of the United States of America* **111**, 11413-11418, doi:10.1073/pnas.1411558111 (2014).

24 Stracy, M., Uphoff, S., Garza de Leon, F. & Kapanidis, A. N. In vivo single-molecule imaging of bacterial DNA replication, transcription, and repair. *FEBS letters* **588**, 3585-3594, doi:10.1016/j.febslet.2014.05.026 (2014).

25 Uphoff, S., Sherratt, D. J. & Kapanidis, A. N. Visualizing protein-DNA interactions in live bacterial cells using photoactivated single-molecule tracking. *Journal of visualized experiments : JoVE*, doi:10.3791/51177 (2014).
