# Supplementary material for: Single-molecule imaging of UvrA and UvrB recruitment to DNA lesions in living Escherichia coli
Source: Nat Commun. 2016 Aug 26;7:12568. doi: 10.1038/ncomms12568 (PMC5007444; doi:10.1038/ncomms12568)
Supplement: Peer review file [file ncomms12568-s2.pdf]

Reviewers' Comments:

Reviewer #1 (Remarks to the Author)

Bacterial nucleotide excision repair is choreographed by the UvrA, UvrB, and the UvrC proteins. Despite well over three decades of structure-function studies of these proteins, significant gaps in our knowledge exist as to how these proteins mediate damage recognition and incision in the milieu of a living cell. This study from an outstanding team uses single molecule tracking PALM to follow PAmCherry-labeled UvrA or UvrB. The authors follow the types of motion and diffusion behaviors of these two proteins in several genetic backgrounds. They also examine the effects of UvrA mutations in the proximal and distal ATP binding sites. The authors first delve into the nature of immobile fraction of UvrA molecules in the absence of UV damage, they speculate that these are molecules searching for damage. They also assume that these immobile UvrA molecules are bound to DNA, but do not give any direct evidence. Showing good co-localization to DNA is important since Grossman and coworkers reported in 1997 (Nucleic Acids Res 25(15):3151-3158), using several approaches including colloidal gold antibody staining and TEM to follow UvrA before and after UV, finding a significant fraction that are membrane bound after UV. The authors report a significant larger fraction (~75% as compared to about 42%) of immobile UvrA molecules after UV damage, suggesting specific binding to UV-induced photoproducts. They rule out increased binding due to interaction with Mfd, the transcription coupling factor. With regard to the motile fraction, after fitting the observed diffusion rates to a double and triple distribution for UvrA and UvrB, respectively, they report that the rates of diffusion 0.31 μm2s-1 and 0.41 μm2s-1, respectively (also see point # 7, below). However, no statistical test was applied as to whether these differences are significant. Based on these small differences they argue that the two proteins rarely interact in solution; which is contrary to 30 years of biochemical, structural and biophysical data on these proteins. As discussed below their pull down experiments are lacking key controls and thus cannot be used as independent evidence for the idea that UvrB only binds to preformed UvrA-DNA complexes. Finally the authors use mutant UvrA proteins defective in either proximal or distal ATPase sites to follow their behavior on and off DNA. They make the interesting and important finding that a proximal ATPase knockout mutant E514A, apparently stops UvrB loading, whereas E858A knocks out distal ATPase but recruits UvrB. Overall this is an important technical advance for the field, but the authors often fail to place their observations into the larger context of the published literature, and in the absence of verification using other approaches , they thus over interpret their findings and try to reach conclusions that cannot be confirmed. Consequently without more extensive experiments as suggested below, authors should be more cautious in their interpretations. To this end, authors should consider the following points in improving the impact of the work:

1. It's not clear from the information provided, how long after UV damage UvrA and UvrB were imaged. This is important as repair of 6-4 photoproducts are more rapid than CPD. How long do the immotile fractions persist in a WT cell? A time course would help confirm know repair rates of UV-induced photoproducts.
2. Page 4 line 10: UvrA is dimeric, do the authors see both molecules, can they show a dual photobleaching profile? While turning on both molecules in the same dimer might be difficult, at sufficient fluences, they should be activate about both monomers in the dimer in ~ 25% of the molecules.
3. If however it is impossible to activate both monomers in a dimer, their calculations need to take into account that they are likely double counting the same molecule, as one monomer will light up followed by another. Its not clear they took this into account.
4. Page 4 line 28: The authors suggest UvrA searches for damage using facilitated diffusion , for which there is no evidence. In fact Kad and coworkers using single molecule analysis of UvrA have shown it to use a 3D mechanism (Mol Cell 37(5):702-13, 2010), and that UvrA does not, in the absence of UvrB, slide on DNA. Furthermore the life time measurements of UvrA complexes in this Kad study are in close agreement to what is reported in this present study - these data should be

clearly mentioned.
5. Their explanation for the 40% statically bound is weak, it is more likely that this reflects the relationship between lifetime of attachment and sampling time. The error on their plots is difficult to rationalize ad they only examined 3 datasets; its not clear how did they arrive at such a low error.
6. It is not clear why the diffusion constants reported for both UvrA and UvrB are so much lower than expected for proteins of the expected mass. Thus either the fitting algorithm is flawed or the proteins are bound with a complex of proteins with a larger mass. Both UvrA and UvrB have been reported to interact with RNA polymerase subunit beta for example.
7. One of the most important points that the authors attempt to make is that UvrA and UvrB have significantly different diffusion constants, and therefore do not diffuse together in solution; fitting was based on data that is over a broad range and its not clear how well the overall fits are, as no r2 values were given. Furthermore since no statistical tests are given (throughout the paper) to show that these distributions are different. Furthermore the triple fit of the UvrB data gives a small populations with 0.41 μm2s-1, is this number really significantly different than diffusion 0.31 μm2s-1 ? An F test should be given to shown that a triple fit is the most appropriate.
8. Since UV produces two types of DNA photoproducts, CPD and 6-4 photoproducts and UvrA has higher affinity for the latter, it might be expected to show different off rates. Can this be seen in the data?
9. DNA photolyase is activated by blue light and it is possible that direct reversal of UV-induced CPD could occur during handling or imaging involved in these experiments. This is not a trivial point as Aziz Sancar's group has shown that DNA photolyase (in the dark) helps to increase UvrA's detection of CPD; thus what effect of DNA photolyase deletion have on the types of motion, diffusion kinetics and off rates?
10. Is there an UV fluency dependence to these fraction of immotile particles, and can this be saturated?
11. It is interesting to note in Figure 1, that in the absence of UvrB, there are significantly more immobile UvrA molecules. This is highly consistent with UvrB's known role to decrease UvrA's non-specific binding activity and increase UvrA's specific binding affinity. This should be mentioned and appropriate papers referenced.
12. UvrA's specificity for damaged versus non-damaged has been shown to not be that large (probably less than an order of magnitude) and has been reported in a number of different studies using several different approaches. If one uses the authors own off rate data (extended data Figure 5) assuming the same on rate than the difference between specific and non-specific binding is only a factor of 4. However since UvrA is expected to dissociate once it has loaded UvrB the off-rate of UvrA in the presence of damage is complicated. The authors should report the off-rate of UvrA in the absence of UvrB. The authors missed an opportunity to fill an important gap on the rates of UvrA dissociation from a lesion with the aid of UvrB.
13. Figure 3: Are these conclusions justified from such a small change with the different mutants and no statistics?
14. Pull down experiments in extended data Figure 4 are not under true equilibrium conditions and are missing several key controls. One key control is whether UvrB loading occurs more efficiently if UvrA is pre-bound to lesion sites VERSUS the rate and extent of loading of UvrB, when DNA is added to UvrAB complexes (in the presence of ATP). Careful time course experiments would reveal which process is more efficient. Also to recapitulate in vivo experiments these experiment should be performed in the presence of excess undamaged DNA. Under these conditions I would predict that the UvrAB complex is much more efficient in loading of UvrB as compared to trying to preload UvrA to damaged sites and then add UvrB . Also length of substrates is an important variable since UvrAB complexes actively move on DNA it might be expected that UvrB loading would be more efficient on a reaction in which the lesion is placed on a long DNA substrate versus a short substrate plus competitive DNA (thus keeping total DNA constant) in the reaction. Furthermore an ATP regenerating system should be used in these experiments as the overall amount of ATP hydrolysis in these type of experiments can be large enough to generate ADP which could bind and inhibit specific steps in damage processing. Finally, the authors do not show the starting amounts of protein in the reactions so a full accounting of the amount of bound versus free protein cannot

be made.
15. Page 9 line 15: UvrA has two UvrB binding sites so a perfectly plausible explanation is that Mfd can share occupation of UvrA.
16. It has been shown in several studies that ATP-γ-S has been shown to increase UvrA's non-specific binding and thus it is not unexpected that mutations is UvrA's proximal ATP binding site causes more DNA binding, even in the presence of UvrB.
17. The structure of the paper with most of the information in the extended figures and supplemental information is cumbersome and unfortunate as it forces the reader to constantly flip back and forth. Perhaps more figures could be incorporated into the main text?
18. The supplementary information appears to have been hastily put together as there are a large number of typos and misspellings.
19. The authors do not do a good job of citing previous literature, and relevant data therein. For example, one of several major omissions is the lack of mention of Smith et al., (J Bacteriol. 2002 Jan;184(2):488-93.), where recruitment to the nucleoid was amply demonstrated.

Reviewer #2 (Remarks to the Author)

UvrA and UvrB play crucial roles in detecting the regions of damaged DNA for the nucleotide excision repair process. According to the accepted model, UvrA and UvrB form stable complexes in solution, which has probably been proposed only based on results of in vitro experiments. In this manuscript, Stracy et al. employed PALM-based single particle tracking and showed interesting experimental results indicating that UvrA alone recognized the damaged region and subsequently recruited UvrB. In addition, they gave evidence by mutagenic analyses that ATP hydrolysis at the proximal binding site of UvrA was required for the recruitment of UvrB. Furthermore, they are proposing that ATP hydrolysis at the distal site triggered UvrA release from pre-incision complex. This study gives new insights of the formation of Uvr complexes that is essential for the DNA repairing by analyzing in living bacterial cells, and this reviewer recognized it an important finding that could be achieved only by employing recently established super-resolution microscopy. The data analyses are fairly reliable and the topic is interesting enough for broad audience of Nature Communications, however, there are several concerns came up to the reviewer, which the authors should be clarify prior to getting a publication.

Major criticisms

1) The author observed two species of UvrA with different diffusion coefficient, and assigned the one with lower diffusion coefficient (D=0.11 µm²/s) as immobile. However, in general, molecular movement with this diffusion coefficient is still significant. How the authors interpret the UvrA molecules bound to the damaged region are still slowly moving?

2) The diffusion coefficient of slowly moving species of UvrA is 0.31 µm²/s, which is definitively smaller than free diffusion (cf. fast diffusion of UvrB as well as K646A mutant of UvrA). How do the authors interpret this slow movement? Literary it is reported that the affinity of UvrA to non-damaged DNA is only 2-3 fold less than damaged DNA [Croteau DL et al. J Biol Chem 281: 26370 - 26381 (2006)]. This reviewer is wondering if the slow movement reflect sliding of UvrA on the surface of DNA? If so, does the lack of the fast component of UvrA indicate that UvrA is exclusively existing on the DNA surface?

3) The slow component of UvrB shows slightly faster movement (D=0.41 µm²/s) than the same conpoment of UvrA, and this movement is for sure not free diffusion, and the author is claiming it is not interaction with UvrA. Does this mean UvrB itself can interact directly to DNA, probably non-damaged regions and slowly moving there? If so, how UvrB interacts to the DNA?

4) How do the authors interpret the presence of small amount of immobile UvrB even under the ΔuvrA condition? Under this condition, UvrB cannot be immobilized due to the recruitment the

damaged DNA region via UvrA.

5) For the binding time analysis, the onset of the binding time should be the time of binding. However, photoactivation of PA-mCherry fused to Uvr proteins binding to the damaged DNA can be experimentally recognized as the onset, and in this case the binding time is underestimated. How do the authors eliminate these molecules from the analysis?

Minor comments

1) Related to the major criticism 1, the proposed model in Fig. 3b should be re-considered in terms of UvrA recruitment from solution at the first stem, as well as release from DNA at the last step. Lack of fast diffusing UvrA species suggests UvrA exist exclusively on DNA surface.

2) The result of E858A, shown in extended Figure 7, is an essential result to draw their proposed mechanism that ATP hydrolysis at the distal site triggered UvrA release from the repair complex. It should be included in Fig. 3 panel b.

3) The end of first paragraph at page 9, "after false-positive damage recognition": This reviewer feels strange to use the term "false-positive". This is probably accumulation of spontaneous DNA damages due to the lack of the NER activity (because of this mutation). As a result, UvrA/B might accumulate at the damaged region without UV illumination, but this is not "false-positive damage recognition".

Reviewer #3 (Remarks to the Author)

This is a fascinating paper, which monitors the mobility of single labelled UvrA and UvrB repair proteins in living bacteria and reveals most unexpected results. The dominant model for damage recognition in bacterial nucleotide excision repair involves a complex of UvrA and UvrB, but here the data show that the two proteins demonstrate quite different behaviour from each other within cells. The authors conclude that UvrB and UvrA rarely form a complex in solution, and propose that damage recognition takes place in a two-step mechanism in which UvrA first finds the damage and then recruits DNA from solution. They also demonstrate differential effects of mutating the two ATPase sites of UvrA, and suggest a model in which coordinated ATP hydrolysis at the two sites recruits UvrB and releases UvrA.

The principal conclusion of this work is revolutionary, and will overthrow a long-held (and textbook-entrenched) model if it proves to be correct. There is a large body of biochemical evidence for UvrAB complexes in vitro, and I think that this paper will trigger a great deal of effort in this field to reconcile that past work with the current findings, and understand the apparent discrepancies. Nonetheless, the work in this manuscript stands up well in its own right, and I think that it is an important and exciting study that deserves rapid exposure in a high profile journal.

There are a few issues that I would raise for consideration prior to publication:

1. An obvious concern with this kind of work is that the proteins are fused to PAmCherry, and this might interfere with their normal intermolecular interactions. This is addressed in this work by performing UV survival assays, in which the fusion proteins can compensate for loss of the wild-type protein. This confirms that the proteins can successfully complete repair, and hence must largely retain function, but have the authors considered the kinetics of repair? i.e. might the PAmCherry fusion proteins have defects that cause damage to be recognised more slowly than in wt cells? On a related, but more minor note, on p30 there is a statement that "The cellular behaviour of [fusion] expression cells changed predictably when cells were exposed to UV light" - What does this mean?

2. It would be very useful to state the total time post-UV required to complete the analysis of protein mobility (I couldn't see a way of working this out from the text). The experiment starts 5 minutes post-UV - if the total sampling time is also multiple minutes then repair will be taking place and SOS induction may be changing UvrA and UvrB concentrations, meaning that the data is

not being collected at a "steady state". If the sampling time is short this would not be a concern.
3. Whilst there is not space in such a short manuscript to review all of the prior work, I feel that the work in reference 22, which investigated the role of the proximal and distal ATPase activities, is being rather overlooked when statements about the functions of the sites remaining elusive are being made (although the paper is cited elsewhere). This paper should certainly be cited in line 20 on p3, and line 19 of p7, and I feel that ideally space should be found to expand on the findings of this earlier paper, which demonstrates differential roles in loading of UvrB, when discussing the findings of the current work.
4. Extended data figure 4 (UvrB recruitment from solution). (i)The figure shows UvrA being added to damaged DNA that was already bound to beads, but the legend and method section indicates that the binding to beads occurred after incubation of DNA with UvrA. (ii) Was a control experiment measuring binding of UvrB in the absence of UvrA? (iii) On p40 the authors state that, to their knowledge, the recruitment of UvrB directly to DNA-bound UvrA has not previously been tested. In fact this has been looked at previously, and in support of the current work reference 7 reports recruitment of UvrB to DNA-bound UvrA in in vitro single molecule experiments with DNA tightropes.
5. Details of strains and plasmids in supplementary information. The details provided were rather sparse, and would not allow the experiments to be repeated. The nature of the kan-linked gene deletions in strains constructed should be explained (e.g. what is the extent of the deletion, are these deletions that have been moved from other strains?). The nature of the PAmCherry fusion constructs should be explained (the text states that UvrA is a C-teminal fusion, but I didn't spot this information for UvrB or Mfd. Were residues lost from the target proteins, or linkers added when making the fusions?). More details of the plasmids used should be given (pZ84-86 states which restriction sites were used, but not the extent of the gene fragment cloned - did the genes retain their own promoters and terminators, and if so to what positions?). The pET28-based plasmids used in section 9 are missing from the table.
6. Typos: P16 line 5 "No damage was introduced" seems to be redundant. Extended DAt Fig1 a: the rows of UV survival spots need strain labels. P36 line 36 "braoder"

Reviewers' comments:

Reviewer #1 (Remarks to the Author):

Bacterial nucleotide excision repair is choreographed by the UvrA, UvrB, and the UvrC proteins. Despite well over three decades of structure-function studies of these proteins, significant gaps in our knowledge exist as to how these proteins mediate damage recognition and incision in the milieu of a living cell. This study from an outstanding team uses single molecule tracking PALM to follow PAmCherry-labeled UvrA or UvrB. The authors follow the types of motion and diffusion behaviors of these two proteins in several genetic backgrounds. They also examine the effects of UvrA mutations in the proximal and distal ATP binding sites. The authors first delve into the nature of immobile fraction of UvrA molecules in the absence of UV damage, they speculate that these are molecules searching for damage. They also assume that these immobile UvrA molecules are bound to DNA, but do not give any direct evidence. Showing good co-localization to DNA is important since Grossman and coworkers reported in 1997 (Nucleic Acids Res 25(15):3151-3158), using several approaches including colloidal gold antibody staining and TEM to follow UvrA before and after UV, finding a significant fraction that are membrane bound after UV.
Indeed, cell fractionation and EM data from Grossman et al. indicated that UvrA is recruited to the membrane after UV exposure. On the other hand, Smith et al., (J Bacteriol. 2002 Jan;184(2):488-93.), as pointed out in point 19 of this review, have shown that UvrA is located in the nucleoid before and after inducing DNA damage in live cells, and show no evidence of recruitment to the membrane. We performed additional experiments and analysis to look at the spatial distribution of UvrA and UvrB: we imaged UvrA and UvrB with stained DNA, and we generated 2D heatmaps of the intracellular distribution of UvrA and UvrB from hundreds of cells and compared this to the distribution of nucleoid associated proteins HU. Our results show that UvrA is strongly associated with the nucleoid region both in the presence and absence of DNA damage, in agreement with data presented by Smith et al. We do not find evidence of a membrane association for UvrA before or after UV exposure. UvrB showed much less association with the nucleoid, consistent with the majority of UvrB diffusing freely. However, exposure to UV caused a relocation of most UvrB to the nucleoid regions, consistent with association to UvrA at damage sites on DNA. These additional data have been added to the manuscript.

The authors report a significant larger fraction (~75% as compared to about 42%) of immobile UvrA molecules after UV damage, suggesting specific binding to UV-induced photoproducts. They rule out increased binding due to interaction with Mfd, the transcription coupling factor. With regard to the motile fraction, after fitting the observed diffusion rates to a double and triple distribution for UvrA and UvrB, respectively, they report that the rates of diffusion 0.31 µm2s-1 and 0.41 µm2s-1, respectively (also see point # 7, below). However, no statistical test was applied as to whether these differences are significant. Based on these small differences they argue that the two proteins rarely interact in solution; which is contrary to 30 years of biochemical, structural and biophysical data on these proteins.
We apologise for not presenting our results clearly enough. We observed that the majority (~60%) of UvrB molecules are fast diffusing (1.2 $\mu m^2 s^{-1}$) in cells, no such population is observed for UvrA. We have now included the two *D** distributions overlaid so that the difference can be observed more clearly. The non-overlapping UvrB population (the majority of molecules) cannot be complexed to

UvrA. There remains a population of slow moving molecules, accounting for 25% of UvrB molecules, however since the fits to this population showed that they were still more mobile than UvrA (0.41 $\mu m^2 s^{-1}$ and 0.31 $\mu m^2 s^{-1}$, respectively) we hypothesised that these may also not reflect complexed UvrA-UvrB. To test this, we performed the same analysis of UvrB in cells lacking UvrA. This showed no change in the abundance of the 0.41 $\mu m^2 s^{-1}$ population. We have rephrased the text to make this clearer. We have also performed significance testing on the abundance and mobility of the 0.41 $\mu m^2 s^{-1}$ population in wt vs Δ*uvrA* cells, which showed no significant difference. We also confirmed that the difference in mobility of the slow moving populations for UvrA and UvrB is significant. We have moved the fitted distribution of D* values of UvrB in Δ*uvrA* cells from the supplement to a main figure so that these details can be more easily verified without referring to the supplement. Finally, we have included additional analysis of the spatial distribution of UvrA and UvrB, which is in agreement with our conclusions (see above).

As discussed below their pull down experiments are lacking key controls and thus cannot be used as independent evidence for the idea that UvrB only binds to preformed UvrA-DNA complexes. Finally the authors use mutant UvrA proteins defective in either proximal or distal ATPase sites to follow their behavior on and off DNA. They make the interesting and important finding that a proximal ATPase knockout mutant E514A, apparently stops UvrB loading, whereas E858A knocks out distal ATPase but recruits UvrB. Overall this is an important technical advance for the field, but the authors often fail to place their observations into the larger context of the published literature, and in the absence of verification using other approaches , they thus over interpret their findings and try to reach conclusions that cannot be confirmed. Consequently without more extensive experiments as suggested below, authors should be more cautious in their interpretations. To this end, authors should consider the following points in improving the impact of the work:

1. It's not clear from the information provided, how long after UV damage UvrA and UvrB were imaged. This is important as repair of 6-4 photoproducts are more rapid than CPD. How long do the immotile fractions persist in a WT cell? A time course would help confirm know repair rates of UV-induced photoproducts.

As detailed in the methods section; PALM acquisition was started < 5 minutes after slides were exposed to UV and imaging takes no more than 15 minutes. We considered performing a time course experiment, however the interpretation of such an experiment would be problematic due to SOS induction. Increased levels of UvrA and UvrB would most likely reduce relative fractions of immobile molecules making impossible to distinguish it from completion of the repair.

2. Page 4 line 10: UvrA is dimeric, do the authors see both molecules, can they show a dual photobleaching profile? While turning on both molecules in the same dimer might be difficult, at sufficient fluences, they should be activate about both monomers in the dimer in ~ 25% of the molecules.

While both fluorophores will eventually be activated and imaged over the course of the acquisition, referring these two stochastic photoactivation events to the same dimer is not possible.

3. If however it is impossible to activate both monomers in a dimer, their calculations need to take into account that they are likely double counting the same molecule, as one monomer will light up followed by another. Its not clear they took this into account.

The analysis does indeed count each dimer as two independent trajectories. We have assumed that in WT conditions essentially ~100% of UvrA molecules are in dimers. The fractions of molecules in each state will therefore remain unchanged, however the absolute numbers of measured trajectories in each state, will be double the expected number of dimers in each state. We have clarified this in the figure 3 legend.

4. Page 4 line 28: The authors suggest UvrA searches for damage using facilitated diffusion , for which there is no evidence. In fact Kad and coworkers using single molecule analysis of UvrA have shown it to use a 3D mechanism (Mol Cell 37(5):702-13, 2010), and that UvrA does not, in the absence of UvrB, slide on DNA.
We agree that we have no evidence that UvrA is using facilitated diffusion, and the statement has been removed.

Furthermore the life-time measurements of UvrA complexes in this Kad study are in close agreement to what is reported in this present study - these data should be clearly mentioned.
We thank the reviewer for pointing out this agreement, and we have included this fact in the text.

5. Their explanation for the 40% statically bound is weak, it is more likely that this reflects the relationship between lifetime of attachment and sampling time. The error on their plots is difficult to rationalize ad they only examined 3 datasets; its not clear how did they arrive at such a low error.
We apologies that the error analysis was not sufficiently explained in the main text. Given the very large n for these assays (typically tens of thousands of molecules), the statistical error in the fitting to the *D** distributions, as determined by bootstrap resampling, is very small. We therefore chose to represent the larger error derived from fitting the *D** distributions from independent experimental repeats of cells grown and imaged separately. The errors quoted on the bar plots therefore represent the standard error of the mean fitted value extracted by fitting to the distribution of D* values for at least 3 independent experimental repeats. Each experimental repeat consists of several movies taken of different fields of view (each representing >50 cells), and comprises many thousands of molecules (see Supplementary figure legends for total numbers of molecules analysed).

Based on our assay, 40% of UvrA molecules do not show displacements greater than expected from the localisation error over the course of the trajectory. In the text we refer to these molecules as immobile. This has been clarified in the text.

6. It is not clear why the diffusion constants reported for both UvrA and UvrB are so much lower than expected for proteins of the expected mass. Thus either the fitting algorithm is flawed or the proteins are bound with a complex of proteins with a larger mass. Both UvrA and UvrB have been reported to interact with RNA polymerase subunit beta for example.
The low observed apparent diffusion of UvrA is a result of transient interactions with DNA. This effect has been observed for several DNA binding proteins, starting with the first *in vivo* studies of LacI by Elf and colleagues. In our laboratories, we have also observed that other DNA binding proteins (RNAP; Stracy 2015, TopoIV, Zawadzki 2015), known to non-specifically bind DNA show lower than expected apparent diffusion coefficient. We note also that the apparent diffusion coefficients reported here is affected by small confined space of cell interior, which we analysed and

commented on in Supplementary Methods; section 7. Additionally, we performed molecular simulations to predict the D* for immobile, mobile and transiently interacting with DNA, UvrA dimers (Fig. 1c).
We now explain this phenomenon in the main text and have added a panel of simulated diffusion for UvrA dimers to Figure 1. This shows the expected apparent diffusion for UvrA dimer stably bound to DNA, interacting transiently (<<15ms) with DNA and freely diffusing in cell interior.

7. One of the most important points that the authors attempt to make is that UvrA and UvrB have significantly different diffusion constants, and therefore do not diffuse together in solution; fitting was based on data that is over a broad range and its not clear how well the overall fits are, as no r2 values were given. Furthermore since no statistical tests are given (throughout the paper) to show that these distributions are different. Furthermore the triple fit of the UvrB data gives a small populations with 0.41 µm2s-1, is this number really significantly different than diffusion 0.31 µm2s-1 ? An F test should be given to shown that a triple fit is the most appropriate.
We have now performed significance testing on the mobility of the slow diffusing populations of UvrA and UvrB. Five independent sets of experimental data were fitted with an unconstrained model with either two species (for UvrA) or three species (for UvrB). The values extracted from these fits were compared between UvrA and UvrB. The difference was significant.
Given that we are using maximum likelihood estimation, we calculated the Bayesian information criterion (BIC) to test the model selection for a two and three species fit to the distribution of UvrB D* values. The three species model had a lower BIC than two species, indicating a better model. A two species fit to the distribution of D* values for UvrB is shown in Fig. S1f, which can be seen to describe the data poorly.

8. Since UV produces two types of DNA photoproducts, CPD and 6-4 photoproducts and UvrA has higher affinity for the latter, it might be expected to show different off rates. Can this be seen in the data?
Our assays could not resolve these two processes; however, we cannot rule out that they take place.

9. DNA photolyase is activated by blue light and it is possible that direct reversal of UV-induced CPD could occur during handling or imaging involved in these experiments. This is not a trivial point as Aziz Sancar's group has shown that DNA photolyase (in the dark) helps to increase UvrA's detection of CPD; thus what effect of DNA photolyase deletion have on the types of motion, diffusion kinetics and off rates?
We deleted the photolyase gene and observed no effect on the behaviour of UvrA in undamaged cells. A modest decrease in immobile UvrA molecules was observed after UV exposure. The new data are included in Fig. 2.

10. Is there an UV fluency dependence to these fraction of immotile particles, and can this be saturated?
Yes, there are fewer immobile molecules at lower UV dosage but further increase of UV dose did not increase the fraction of immobile particle any further. Therefore, 50J/m^2, as used, is a saturation level for proportion of UvrA/B involved in the repair. This dose was chosen in order to prevent completion of repair before completion of the experiment (typically between 5 and 20 minutes after UV exposure).

11. It is interesting to note in Figure 1, that in the absence of UvrB, there are significantly more immobile UvrA molecules. This is highly consistent with UvrB's known role to decrease UvrA's non-specific binding activity and increase UvrA's specific binding affinity. This should be mentioned and appropriate papers referenced.

We have now mentioned this in the text and have included the relevant references.

12. UvrA's specificity for damaged versus non-damaged has been shown to not be that large (probably less than an order of magnitude) and has been reported in a number of different studies using several different approaches. If one uses the authors own off rate data (extended data Figure 5) assuming the same on rate than the difference between specific and non-specific binding is only a factor of 4. However since UvrA is expected to dissociate once it has loaded UvrB the off-rate of UvrA in the presence of damage is complicated. The authors should report the off-rate of UvrA in the absence of UvrB. The authors missed an opportunity to fill an important gap on the rates of UvrA dissociation from a lesion with the aid of UvrB.

We agree that the off-rate of UvrA is complicated by action of UvrB. To address if UvrB availability is a rate-limiting step in UvrA off-rate, we now include binding times for UvrA in the presence of UvrB overexpression. Our assay is not accurate enough to accurately measure very long binding times as observed for UvrA after UV exposure in the absence of UvrB, therefore we decided to not include this results in the text.

13. Figure 3: Are these conclusions justified from such a small change with the different mutants and no statistics?

We concede that the significance of the further increase of immobile $UvrA^{K37A}$ after UV exposure is not clear from the bar graph alone. We have therefore performed a two-sample t-test on the fraction of immobile $UvrA^{K37A}$ before and after UV exposure, which showed the effect to be significant. This has been added to the main text.

14. Pull down experiments in extended data Figure 4 are not under true equilibrium conditions and are missing several key controls. One key control is whether UvrB loading occurs more efficiently if UvrA is pre-bound to lesion sites VERSUS the rate and extent of loading of UvrB, when DNA is added to UvrAB complexes (in the presence of ATP). Careful time course experiments would reveal which process is more efficient. Also to recapitulate in vivo experiments these experiment should be performed in the presence of excess undamaged DNA. Under these conditions I would predict that the UvrAB complex is much more efficient in loading of UvrB as compared to trying to preload UvrA to damaged sites and then add UvrB. Also length of substrates is an important variable since UvrAB complexes actively move on DNA it might be expected that UvrB loading would be more efficient on a reaction in which the lesion is placed on a long DNA substrate versus a short substrate plus competitive DNA (thus keeping total DNA constant) in the reaction. Furthermore an ATP regenerating system should be used in these experiments as the overall amount of ATP hydrolysis in these type of experiments can be large enough to generate ADP which could bind and inhibit specific steps in damage processing. Finally, the authors do not show the starting amounts of protein in the reactions so a full accounting of the amount of bound versus free protein cannot be made.

We agree that the reviewer’s idea for a time course experiment comparing the loading efficiency of UvrB to damaged DNA by pre-loaded UvrA versus UvrA and UvrB together. We have now performed

a time course experiments of UvrB loading under these conditions. In this experiment excess undamaged DNA was also added as suggested. At early time points we observed significantly higher loading of UvrB by pre-loaded UvrA compared to adding pre-mixed UvrA and UvrB together, confirming more efficient loading of UvrB by preloaded UvrA, in agreement with our hypothesis. This data has been added to the Fig 5.
Unfortunately, an ATP regeneration system is incompatible with our assay due to interactions with the beads. We note that Oh and Grossman (PNAS 1987) found that ATP regeneration system has no effect on UvrAB helicase activity in their *in vitro* assays.
We agree that looking at the target search of UvrA in locating damage by changing the lengths of DNA substrate would be interesting to study, but it is beyond the scope of this work.

15. Page 9 line 15: UvrA has two UvrB binding sites so a perfectly plausible explanation is that Mfd can share occupation of UvrA.
We still believe this is a useful point to stress, since there is still considerable debate in the field as to the stoichiometry of the UvrA-UvrB complex, and much of the literature states that the complex is $UvrA_2UvrB_2$, which would be incompatible with Mfd binding (Wagner 2010, Pakotiprapha 2012).

16. It has been shown in several studies that ATP-γ-S has been shown to increase UvrA's non-specific binding and thus it is not unexpected that mutations is UvrA's proximal ATP binding site causes more DNA binding, even in the presence of UvrB.
We thank the reviewer for pointing this out and we have mentioned this in the text.

17. The structure of the paper with most of the information in the extended figures and supplemental information is cumbersome and unfortunate as it forces the reader to constantly flip back and forth. Perhaps more figures could be incorporated into the main text?
We agree and have included crucial results from the supplementary material into the main figures.

18. The supplementary information appears to have been hastily put together as there are a large number of typos and misspellings.
We have thoroughly copy edited the supplementary text.

19. The authors do not do a good job of citing previous literature, and relevant data therein. For example, one of several major omissions is the lack of mention of Smith et al., (J Bacteriol. 2002 Jan;184(2):488-93.), where recruitment to the nucleoid was amply demonstrated.
We thank the reviewer for pointing out this reference, and we have now included it. In addition, we have expanded several sections of the text to put our findings in the context of the previously published work.

Reviewer #2 (Remarks to the Author):

UvrA and UvrB play crucial roles in detecting the regions of damaged DNA for the nucleotide excision repair process. According to the accepted model, UvrA and UvrB form stable complexes in solution, which has probably been proposed only based on results of in vitro experiments. In this manuscript, Stracy et al. employed PALM-based single particle tracking and showed interesting experimental results indicating that UvrA alone recognized the damaged region and subsequently recruited UvrB.

In addition, they gave evidence by mutagenic analyses that ATP hydrolysis at the proximal binding site of UvrA was required for the recruitment of UvrB. Furthermore, they are proposing that ATP hydrolysis at the distal site triggered UvrA release from pre-incision complex. This study gives new insights of the formation of Uvr complexes that is essential for the DNA repairing by analyzing in living bacterial cells, and this reviewer recognized it an important finding that could be achieved only by employing recently established super-resolution microscopy. The data analyses are fairly reliable and the topic is interesting enough for broad audience of Nature Communications, however, there are several concerns came up to the reviewer, which the authors should be clarify prior to getting a publication.

Major criticisms

1) The author observed two species of UvrA with different diffusion coefficient, and assigned the one with lower diffusion coefficient (D=0.11 $\mu m^2/s$) as immobile. However, in general, molecular movement with this diffusion coefficient is still significant. How the authors interpret the UvrA molecules bound to the damaged region are still slowly moving?
The non-zero mean apparent diffusion coefficient of immobile molecules in our assay is due to the non-zero localisation uncertainty in each measurement, σ_{loc}, which manifests itself as a positive offset in the *x* axis of the distribution of apparent diffusion coefficients of $\sigma_{loc}^2/\Delta t$ (Michalet & Berglund, 2012). This is explained in the Supplementary Methods. For clarity we have also now included this explanation in the main text.

2) The diffusion coefficient of slowly moving species of UvrA is 0.31 $\mu m^2/s$, which is definitively smaller than free diffusion (cf. fast diffusion of UvrB as well as K646A mutant of UvrA). How do the authors interpret this slow movement? Literary it is reported that the affinity of UvrA to non-damaged DNA is only 2-3 fold less than damaged DNA [Croteau DL et al. J Biol Chem 281: 26370 - 26381 (2006)]. This reviewer is wondering if the slow movement reflect sliding of UvrA on the surface of DNA? If so, does the lack of the fast component of UvrA indicate that UvrA is exclusively existing on the DNA surface?
We have no evidence to say that UvrA is sliding on DNA (see our response to reviewer 1 point 4). Our explanation of slowly moving species is that this population represents molecules undergoing transient non-specific interactions with DNA as well as intervals of 3D diffusion occurring on a timescale shorter than our observation time, resulting in the observed diffusion being slower than expected. This is very similar to the behaviour of other DNA binding proteins imaged at similar exposure times (Bakshi 2012, Stracy 2015, Elf 2006, Zawadzki 2015). To explain this phenomenon, we included simulations of UvrA diffusion inside cell in Figure 1, and extended main text for clarity.

3) The slow component of UvrB shows slightly faster movement (D=0.41 $\mu m^2/s$) than the same conpoment of UvrA, and this movement is for sure not free diffusion, and the author is claiming it is not interaction with UvrA. Does this mean UvrB itself can interact directly to DNA, probably non-damaged regions and slowly moving there? If so, how UvrB interacts to the DNA?
It is a puzzling result and we do not have a satisfactory explanation for it. Perhaps UvrB transiently interact with regions of ssDNA as reported by Wang at al. JBC Vol. 281, NO. 22, pp. 15227–15237.

4) How do the authors interpret the presence of small amount of immobile UvrB even under the ΔuvrA condition? Under this condition, UvrB cannot be immobilized due to the recruitment the damaged DNA region via UvrA.

We can only speculate that UvrB also interacts with other protein partners (UvrC, UvrD) independently of UV damage. Importantly, in Δ*uvrA* cells, this fraction remains unchanged before and after exposure to UV.

5) For the binding time analysis, the onset of the binding time should be the time of binding. However, photoactivation of PA-mCherry fused to Uvr proteins binding to the damaged DNA can be experimentally recognized as the onset, and in this case the binding time is underestimated. How do the authors eliminate these molecules from the analysis?

Stochastic photoactivation of molecules before or during binding events does not influence our measurement, because the observed binding times follow an exponential distribution and are therefore memoryless. This means that the probability of a molecule dissociating is not dependent on duration of binding prior to observation. I.e. the probability, *X*, of dissociation *s* seconds after time point *t*, is identical to the probability of dissociation after *s* seconds from time point 0 (protein binding): $P(X > t+s \mid X > t) = P(X > s)$.

Minor comments

1) Related to the major criticism 1, the proposed model in Fig. 3b should be re-considered in terms of UvrA recruitment from solution at the first stem, as well as release from DNA at the last step. Lack of fast diffusing UvrA species suggests UvrA exist exclusively on DNA surface.

We modified the cartoon representing proposed model and added explanation that UvrA undergoes transient non-specific interactions with DNA during its search process. However, we do not think that the statement “exist exclusively on DNA surface” can be justified.

2) The result of E858A, shown in extended Figure 7, is an essential result to draw their proposed mechanism that ATP hydrolysis at the distal site triggered UvrA release from the repair complex. It should be included in Fig. 3 panel b.

Thank you for this suggestion; we have included this data in the main text Fig. 6.

3) The end of first paragraph at page 9, "after false-positive damage recognition": This reviewer feels strange to use the term "false-positive". This is probably accumulation of spontaneous DNA damages due to the lack of the NER activity (because of this mutation). As a result, UvrA/B might accumulate at the damaged region without UV illumination, but this is not "false-positive damage recognition".

We agree that accumulation of unrepairable spontaneous damage may account for much of the increase in immobile UvrA^{E858A} molecules. We have re-written the text to reflect this.

Reviewer #3 (Remarks to the Author):

This is a fascinating paper, which monitors the mobility of single labelled UvrA and UvrB repair

proteins in living bacteria and reveals most unexpected results. The dominant model for damage recognition in bacterial nucleotide excision repair involves a complex of UvrA and UvrB, but here the data show that the two proteins demonstrate quite different behaviour from each other within cells. The authors conclude that UvrB and UvrA rarely form a complex in solution, and propose that damage recognition takes place in a two-step mechanism in which UvrA first finds the damage and then recruits DNA from solution. They also demonstrate differential effects of mutating the two ATPase sites of UvrA, and suggest a model in which coordinated ATP hydrolysis at the two sites recruits UvrB and releases UvrA.

The principal conclusion of this work is revolutionary, and will overthrow a long-held (and textbook-entrenched) model if it proves to be correct. There is a large body of biochemical evidence for UvrAB complexes in vitro, and I think that this paper will trigger a great deal of effort in this field to reconcile that past work with the current findings, and understand the apparent discrepancies. Nonetheless, the work in this manuscript stands up well in its own right, and I think that it is an important and exciting study that deserves rapid exposure in a high profile journal.

There are a few issues that I would raise for consideration prior to publication:

We thank the reviewer for their kind assessment of our work.

1. An obvious concern with this kind of work is that the proteins are fused to PAmCherry, and this might interfere with their normal intermolecular interactions. This is addressed in this work by performing UV survival assays, in which the fusion proteins can compensate for loss of the wild-type protein. This confirms that the proteins can successfully complete repair, and hence must largely retain function, but have the authors considered the kinetics of repair? i.e. might the PAmCherry fusion proteins have defects that cause damage to be recognised more slowly than in wt cells?

We cannot exclude the possibility that the rates of the repair are affected by the presence of the fusion, hence the observed binding times could be overestimates of true rates. However, observed binding times are consistent with some earlier results; binding time for UvrA to non-damaged DNA was reported to be ~7s (Kad et al. 2010) which is longer than our ~3s. Furthermore, the Goosen Lab (Malta et al. 2007) has shown that fusions of UvrB to fluorescent protein (GFP or YFP, which are very similar in architecture to PAmCherry used in our study) do not affect the rate of incision.

On a related, but more minor note, on p30 there is a statement that "The cellular behaviour of [fusion] expression cells changed predictably when cells were exposed to UV light" - What does this mean?

We meant that both UvrA and UvrB become immobilised on DNA after DNA damage is induced, as expected from the increased number of molecules repairing damaged DNA. Failure to respond to UV exposure would have indicated significant problems with the fusion functionality. We agree that this was not clear from our phrasing, and we have removed this sentence.

2. It would be very useful to state the total time post-UV required to complete the analysis of protein mobility (I couldn't see a way of working this out from the text). The experiment starts 5 minutes post-UV - if the total sampling time is also multiple minutes then repair will be taking place and SOS induction may be changing UvrA and UvrB concentrations, meaning that the data is not being collected at a "steady state". If the sampling time is short this would not be a concern.

Typical imaging takes ~5 minutes, and no more than two movies were recorded from each individual slide. Therefore, the sampling time is between 5 and 15 minutes after UV exposure – we clarified this in the main text. We note, that possible reduction in the fraction of immobile molecules as a

result of ongoing repair or induction of SOS response would underestimate the results without affecting our conclusions.

3. Whilst there is not space in such a short manuscript to review all of the prior work, I feel that the work in reference 22, which investigated the role of the proximal and distal ATPase activities, is being rather overlooked when statements about the functions of the sites remaining elusive are being made (although the paper is cited elsewhere). This paper should certainly be cited in line 20 on p3, and line 19 of p7, and I feel that ideally space should be found to expand on the findings of this earlier paper, which demonstrates differential roles in loading of UvrB, when discussing the findings of the current work.
We agree with the reviewer, and have included reference 22, which laid important groundwork for the cooperative nature of the two ATP sites and we have expanded the text to include details of this previous work.

4. Extended data figure 4 (UvrB recruitment from solution). (i)The figure shows UvrA being added to damaged DNA that was already bound to beads, but the legend and method section indicates that the binding to beads occurred after incubation of DNA with UvrA. (ii) Was a control experiment measuring binding of UvrB in the absence of UvrA? (iii) On p40 the authors state that, to their knowledge, the recruitment of UvrB directly to DNA-bound UvrA has not previously been tested. In fact this has been looked at previously, and in support of the current work reference 7 reports recruitment of UvrB to DNA-bound UvrA in in vitro single molecule experiments with DNA tightropes.
We thank the reviewer for highlighting this key finding in reference 7. We have expanded the text to include this. We added a control gel to the supplementary figure 4 showing that lack of UvrA prevents loading of UvrB onto damaged DNA. Additionally, we have expanded our *in vitro* work and moved previous and new results into main text.

5. Details of strains and plasmids in supplementary information. The details provided were rather sparse, and would not allow the experiments to be repeated. The nature of the kan-linked gene deletions in strains constructed should be explained (e.g. what is the extent of the deletion, are these deletions that have been moved from other strains?). The nature of the PAmCherry fusion constructs should be explained (the text states that UvrA is a C-teminal fusion, but I didn't spot this information for UvrB or Mfd. Were residues lost from the target proteins, or linkers added when making the fusions?). More details of the plasmids used should be given (pZ84-86 states which restriction sites were used, but not the extent of the gene fragment cloned - did the genes retain their own promoters and terminators, and if so to what positions?). The pET28-based plasmids used in section 9 are missing from the table.
We have expanded the details on strains and plasmids to address these points.

6. Typos: P16 line 5 "No damage was introduced" seems to be redundant. Extended DAt Fig1 a: the rows of UV survival spots need strain labels. P36 line 36 "braoder"
We have removed and corrected these typos.

Reviewers' Comments:

Reviewer #1 (Remarks to the Author)

This revised manuscript examines the motion of UvrA and UvrB in living E.coli cells before and after UV irradiation using state-of-the art fluorescence microscopic approaches. This is an important breakthrough in the field and as such makes an important contribution. This new version is largely rewritten, and much of the discussion of the data previously in the supplement is now included in the main body of the text. The authors have done an admirable job of dealing with the extensive concerns raised in the first review; in many cases doing more experiments. The work-up of the UvrA ATP binding mutants is an important strength of this study. The new manuscript has been greatly strengthened by the process of review and revision. However, not all the concerns raised were dealt with in a satisfactory manner, and thus some problems persist. Most importantly, the authors are trying to argue that UvrA binds first and then recruits UvrB to the damaged site, versus the current paradigm that UvrA and UvrB form a complex in solution which then interrogates DNA for lesions. I believe they have over interpreted their single molecule data, and their biochemical experiments on loading of UvrB while supporting their premise have overall poor binding and as described below (point 5) could be problematic without an ATP regenerating system. Thus, it would be a concern if the authors do not soften or eliminate this point in the final version.

Specific comments:

1. The two step recognition mechanism is not fully supported by the data presented, thus, lines 6-8 on page 2 in the abstract, and lines 27-29, page 3 in the introduction, and lines 1-18 page 11 need to be greatly softened or eliminated. The authors simply have over interpreted their data. In fact in the original concerns raised: "Point 11. It is interesting to note in Figure 1, that in the absence of UvrB, there are significantly more immobile UvrA molecules. This is highly consistent with UvrB's known role to decrease UvrA's nonspecific binding activity and increase UvrA's specific binding affinity. This should be mentioned and appropriate papers referenced. While they have given more citations". I struggle to see the difference between 60% and 72% in the ΔuvrB. In fact, here you can see that if you compare UvrA immobility between ΔuvrB and wt you see that by removing UvrB a lot more of the UvrA is now free. Surely this suggests that they are working together in undamaged DNA binding. This concern still remains unaddressed, for statements that suggest differences between empirical observations a valid statistical test is imperative. These have once again been omitted. For example is a D of 0.41 μm2s-1 really statistically different than 0.31 μm2s-1?

Furthermore UvrB's known role is to decrease non-specific binding of UvrA and increase specific binding of UvrB - this has been shown by multiple groups using multiple assays. Thus, in vivo it is expected that UvrAB complexes will be very transient on undamaged DNA - it is not clear they can catch these transient binding events.

2. Page 5 lines 8-14: Their data does not land on one side of their argument regarding UvrA binding first or UvrAB binding or the other.

3. The amount of photobleaching versus true dissociation is huge (supplementary Figure 3), what is the total n of particles and what fraction of particles undergo photobleaching versus true dissociation. Surely the authors could simply eliminate photobleached particles from the life-time measurements, unless of course the number of true dissociators is too low.

4. Page 6, lines 5-8: "Therefore, UvrA can still detect and bind damaged DNA even in the absence of UvrB. This result agrees with previous in vitro observations that UvrA is able to discriminate damaged and undamaged DNA independently of UvrB (ref 6,8,17,29).: This argument is not valid in vivo where the amount of non-damaged DNA is in great excess over damaged DNA, the level of damage discrimination, i.e. specific versus non-specific binding of UvrA to DNA is just not good enough in the absence of UvrB to be able to rapidly and efficient recognize damage in vivo. The authors need to do these calculations based on UvrA's poor discrimination, the amount of DNA, the number of UvrA molecules and the off rates.

5. Bottom of page 8 and page 9, biochemical experiments. The addition of the supplemental data is very helpful and its apparent that the total amount of protein binding versus the available protein is probably 10% for UvrB and less than that for UvrA. In Figure 5 a, the bar graph Y-axis is very miss-leading as the authors state, % of UvrB loaded and set no competitor to 100%. But in fact this is probably only 10% of total protein - this is not clear in the legend. Thus it is not clear how to interpret these data when only a small fraction is actually binding. Also it would seem based on the supplemental information that an ATP regenerating system was not used in these experiments. One worries that the amount of UvrAB's combined ATPase activity could be such to increase the levels of ADP sufficient to cause dissociation of UvrB complexes.

Reviewer #2 (Remarks to the Author)

Please refer the attachment.

Reviewer #3 (Remarks to the Author)

In their rebuttal letter the authors' satisfactorily address all of the issues that I raised in my initial review. Importantly, given the potentially controversial nature of the conclusions of this work, I feel that the revised paper now does a good job of placing the findings in the context of prior work.

In their response to my "point 2" (time/duration of sampling, and relationship to repair/SOS induction) the authors state that sampling duration is between 5 and 15 minutes, and that this has been clarified in the main text. I did not notice this clarification when reading the revised manuscript or the supp info, and have not been able to find it using text searches. I think that this is an important issue, and clear statement of sampling timing/duration is essential for interpretation of the results (I note that this was also point 1 of referee #1's comments). I suggest that the editor check that this information is present before publication.

Reviewers' comments:

Reviewer #1 (Remarks to the Author):

This revised manuscript examines the motion of UvrA and UvrB in living E.coli cells before and after UV irradiation using state-of-the art fluorescence microscopic approaches. This is an important breakthrough in the field and as such makes an important contribution. This new version is largely rewritten, and much of the discussion of the data previously in the supplement is now included in the main body of the text. The authors have done an admirable job of dealing with the extensive concerns raised in the first review; in many cases doing more experiments. The work-up of the UvrA ATP binding mutants is an important strength of this study. The new manuscript has been greatly strengthened by the process of review and revision. However, not all the concerns raised were dealt with in a satisfactory manner, and thus some problems persist. Most importantly, the authors are trying to argue that UvrA binds first and then recruits UvrB to the damaged site, versus the current paradigm that UvrA and UvrB form a complex in solution which then interrogates DNA for lesions. I believe they have over interpreted their single molecule data, and their biochemical experiments on loading of UvrB while supporting their premise have overall poor binding and as described below (point 5) could be problematic without an ATP regenerating system. Thus, it would be a concern if the authors do not soften or eliminate this point in the final version.

Specific comments:
1. The two step recognition mechanism is not fully supported by the data presented, thus, lines 6-8 on page 2 in the abstract, and lines 27-29, page 3 in the introduction, and lines 1-18 page 11 need to be greatly softened or eliminated. The authors simply have over interpreted their data.

Our data is very difficult to reconcile with the current paradigm, in which UvrA and UvrB form a complex in solution which then interrogates DNA for lesions:

- In undamaged cells the majority of UvrB are fast diffusing and are not complexed with UvrA.
- In undamaged cells the slow diffusing population of UvrB and most immobile UvrB are not dependent on the presence of UvrA, indicating these UvrB molecules are not complexed with UvrA.
- In undamaged cells UvrA molecules are mainly located in the nucleoid region, whereas UvrB are located throughout the cell volume.
- UvrB relocates to the nucleoid region only after UV treatment.
- The number of immobile UvrA molecules drastically increases after UV treatment, in wt and in uvrB- cells, showing that UvrA can locate damage alone.
- UV exposure results in a large increase in the number of immobile UvrB molecules, and a large decrease in the fast diffusing population, but little change to the slow moving population, consistent with direct recruitment from solution to DNA.
- Overexpression of UvrA resulted in the majority of UvrB being immobile on DNA, but we found no evidence for the generation of any mobile UvrA-UvrB complexes.
- The in vitro competition assay shows that UvrA can efficiently recruit UvrB to DNA damage directly from solution.
- The time course experiment shows that UvrA preloaded onto damaged DNA recruits UvrB more efficiently than when UvrA-UvrB complexes are allowed to form in solution.
- Previous work by others has shown that UvrA can locate damage independently of UvrB in vitro.

We believe that these results are best described by a two-step model, and we would therefore like to have the opportunity to present this model to the scientific community. We do not believe that this is an over interpretation of the results. We made all efforts to present our data clearly and put them in the context of previous reports and models. This should allow the scientific community to evaluate our results in light of their own findings in order to further improve our understanding of NER.

In fact in the original concerns raised: "Point 11. It is interesting to note in Figure 1, that in the absence of UvrB, there are significantly more immobile UvrA molecules. This is highly consistent with UvrB's known role to decrease UvrA's nonspecific binding activity and increase UvrA's specific binding affinity. This should be mentioned and appropriate papers referenced.

As detailed in the previous response, this has been added to the revised manuscript and the appropriate papers mentioned (lines 11-12 page 5).

While they have given more citations". I struggle to see the difference between 60% and 72% in the ΔuvrB. In fact, here you can see that if you compare UvrA immobility between ΔuvrB and wt you see that by removing UvrB a lot more of the UvrA is now free. Surely this suggests that they are working together in undamaged DNA binding.

As detailed in the previous response and the previously revised manuscript, we performed statistical analysis, which showed this difference to be significant (lines 3-6 page 6).

This concern still remains unaddressed, for statements that suggest differences between empirical observations a valid statistical test is imperative. These have once again been omitted. For example is a D of 0.41 µm2s-1 really statistically different than 0.31 µm2s-1?

As detailed in the previous response and the previously revised manuscript, we performed statistical analysis, which showed this difference to be significant (lines 9-12 page 7).

Furthermore UvrB's known role is to decrease non-specific binding of UvrA and increase specific binding of UvrB - this has been shown by multiple groups using multiple assays. Thus, in vivo it is expected that UvrAB complexes will be very transient on undamaged DNA - it is not clear they can catch these transient binding events.

Transient DNA interactions (shorter than the exposure time of 15ms) result in a slower mobility than expected for free diffusion, as observed for UvrA (and other DNA binding proteins, see Elf et al, Science 2007). Since we observe that almost none of the slowly diffusing UvrB molecules are dependent on UvrA in wt cells, we conclude that these do not represent UvrA-UvrB complexes.

2. Page 5 lines 8-14: Their data does not land on one side of their argument regarding UvrA binding first or UvrAB binding or the other.

We agree and note that no such interpretation is made in the text.

3. The amount of photobleaching versus true dissociation is huge (supplementary Figure 3), what is the total n of particles and what fraction of particles undergo photobleaching versus true dissociation. Surely the authors could simply eliminate photobleached particles from the life-time measurements, unless of course the number of true dissociators is too low.

The photobleaching is accounted for as described in the methods and supplementary methods sections. Even if it was possible to simply remove photobleached molecules from the analysis, this would not give the correct estimate for binding times, because this process would generate a sampling bias in which only short binding events remain in the dataset. Instead, the correct approach is to keep all molecule events in the analysis. As described in Uphoff et al. (PNAS 2013), the observed probability of molecule disappearance is the product of the probabilities of bleaching and unbinding, because these processes are independent.

4. Page 6, lines 5-8: "Therefore, UvrA can still detect and bind damaged DNA even in the absence of UvrB. This result agrees with previous in vitro observations that UvrA is able to discriminate damaged and undamaged DNA independently of UvrB (ref 6,8,17,29).: This argument is not valid in vivo where the amount of non-damaged DNA is in great excess over damaged DNA, the level of damage discrimination, i.e. specific versus non-specific binding of UvrA to DNA is just not good enough in the absence of UvrB to be able to rapidly and efficient recognize damage in vivo. The authors need to do these calculations based on UvrA's poor discrimination, the amount of DNA, the number of UvrA molecules and the off rates.

The UvrA discrimination between damaged and undamaged DNA was never tested in vivo. Nevertheless, we observed that the fraction of immobile UvrA increases after inducing DNA damage even in the absence of UvrB, showing that UvrA can locate damage sites without UvrB (Fig.2a). It is currently not possible to discriminate specific vs non-specific binding in vivo.

5. Bottom of page 8 and page 9, biochemical experiments. The addition of the supplemental data is very helpful and its apparent that the total amount of protein binding versus the available protein is probably 10% for UvrB and less than that for UvrA. In Figure 5 a, the bar graph Y-axis is very miss-leading as the authors state, % of UvrB loaded and set no competitor to 100%. But in fact this is probably only 10% of total protein - this is not clear in the legend. Thus it is not clear how to interpret these data when only a small fraction is actually binding.

We have clarified this in the figure legend.

Also it would seem based on the supplemental information that an ATP regenerating system was not used in these experiments. One worries that the amount of UvrAB's combined ATPase activity could be such to increase the levels of ADP sufficient to cause dissociation of UvrB complexes.

As stated in the previous response, an ATP regeneration system is incompatible with our assay due to interactions with the beads. We are confident that 120nM of UvrA will not significantly affect ATP/ADP ratio; since the known ATPase activity of UvrA is ~100ATP/monomer/minute (JBC 1991) and the longest incubation time was 10 minutes. Using these values, the maximum expected change in ATP concentration is from 1mM to ~0.9mM during the course of the experiment (we used 10000 ATP molecules per UvrA monomer). We note that Oh and Grossman (PNAS 1987) found that an ATP

regeneration system had no effect on the helicase activity of UvrAB in their *in vitro* assays, and we use much shorter incubation times than in work cited above.

Reviewer #2 (Remarks to the Author):

This reviewer recognized that the authors addressed most of the criticisms from this reviewer properly. Only minor criticism remains related to the previous major criticism 5, please refer the comments written in red.
5) For the binding time analysis, the onset of the binding time should be the time of binding. However, photoactivation of PA-mCherry fused to Uvr proteins binding to the damaged DNA can be experimentally recognized as the onset, and in this case the binding time is underestimated. How do the authors eliminate these molecules from the analysis?
Stochastic photoactivation of molecules before or during binding events does not influence our measurement, because the observed binding times follow an exponential distribution and are therefore memoryless. This means that the probability of a molecule dissociating is not dependent on duration of binding prior to observation. I.e. the probability, X, of dissociation s seconds after time point t, is identical to the probability of dissociation after s seconds from time point 0 (protein binding): $P(X > t+s \mid X > t) = P(X > s)$.
In this case, the value is “time constant of the off event” but not “dwell time”.

We replaced this in the text accordingly.

Reviewer #3 (Remarks to the Author):

In their rebuttal letter the authors' satisfactorily address all of the issues that I raised in my initial review. Importantly, given the potentially controversial nature of the conclusions of this work, I feel that the revised paper now does a good job of placing the findings in the context of prior work.

In their response to my "point 2" (time/duration of sampling, and relationship to repair/SOS induction) the authors state that sampling duration is between 5 and 15 minutes, and that this has been clarified in the main text. I did not notice this clarification when reading the revised manuscript or the supp info, and have not been able to find it using text searches. I think that this is an important issue, and clear statement of sampling timing/duration is essential for interpretation of the results (I note that this was also point 1 of referee #1's comments). I suggest that the editor check that this information is present before publication.

We apologise for this mistake. We have now added this to the methods section in the main text and the supplement.

Reviewers' Comments:

Reviewer #1 (Remarks to the Author)

This is the second revision of a manuscript following single molecules of UvrA and UvrB in E.coli before and after DNA damage. The previous version was much improved over the original submission, and this newly revised manuscript is mostly a rebuttal of the previous concerns. The authors have given counter arguments to the concerns. Ultimately this study will be shown correct or incorrect by subsequent research from other teams using different approaches. Thus, due to the innovative nature of this work and this being the first study to track these molecules at the single molecule level in living E.coli, it is important to get this work out to the community.
